# Psychosocial and pharmacologic interventions for methamphetamine addiction: protocol for a scoping review of the literature

**DOI:** 10.1186/s13643-020-01499-z

**Published:** 2020-10-24

**Authors:** C. Hamel, K. Corace, M. Hersi, D. Rice, M. Willows, P. Macpherson, B. Sproule, J. Flores-Aranda, G. Garber, L. Esmaeilisaraji, B. Skidmore, A. Porath, R. Ortiz Nunez, B. Hutton

**Affiliations:** 1 Center for Practice Changing Research, Ottawa Hospital Research Institute, The Ottawa Hospital, General Campus, 501 Smyth Road, Box 201b, Ottawa, Ontario K1H 8 L6 Canada; 2 Substance Use and Concurrent Disorders Program, The Royal Ottawa Mental Health Centre, Ottawa, Ontario Canada; 3 Department of Psychiatry, Faculty of Medicine, University of Ottawa, Ottawa, Ontario Canada; 4 Institute of Mental Health Research, University of Ottawa, Ottawa, Ontario Canada; 5 Department of Psychology, McGill University, Montreal, Quebec, Canada; 6 Department of Family Medicine, Faculty of Medicine, University of Ottawa, Ottawa, ON Canada; 7 Division of Infectious Diseases, The Ottawa Hospital, Ottawa, ON Canada; 8 Faculty of Medicine, University of Ottawa, Ottawa, ON Canada; 9 Department of Pharmacy, Centre for Addiction and Mental Health, Toronto, ON Canada; 10 Leslie Dan Faculty of Pharmacy and Department of Psychiatry, University of Toronto, Toronto, ON Canada; 11 Université du Quebec, Montreal, QC Canada; 12 School of Epidemiology, Public Health and Preventive Medicine, University of Ottawa, Ottawa, ON Canada; 13 Public Health Ontario, Toronto, ON Canada; 14 Canadian Center on Substance Use and Addiction, Ottawa, ON Canada; 15 Max Ottawa, Ottawa, ON Canada

**Keywords:** Methamphetamine, Methamphetamine addiction, Methamphetamine use disorder, Psychosocial treatment, Pharmacologic treatment, Scoping review, Knowledge synthesis

## Abstract

**Background:**

Methamphetamine use and harms are rising rapidly. Management of patients with methamphetamine use disorder (MUD) and problematic methamphetamine use (PMU) is challenging, with no clearly established best approach; both psychosocial and pharmacologic interventions have been described. Furthermore, given the diversity of individuals that use methamphetamines, there is a need to assess evidence for treatments for subgroups including youths; gay, bisexual, and other men who have sex with men; individuals with mental health comorbidities; and individuals in correction services. Establishing awareness of the messages regarding treatment from recent clinical practice guidelines (CPG) in the field is also of value.

The first study objective will be to establish a greater understanding of the methods, populations, and findings of controlled studies for psychosocial and pharmacologic treatments for MUD and PMU. Investigation of this information can help establish the potential for advanced syntheses of the evidence (such as network meta-analysis) to compare therapies for this condition and to identify gaps related to key populations where more primary research is needed. Summarizing the recommendations regarding treatment of MUD/PMU from recent CPGs and systematic reviews will be an important secondary objective.

**Methods:**

A scoping review will be performed. Using the OVID platform, MEDLINE, Embase, PsycINFO, and relevant Cochrane databases from EBM Reviews will be searched (from databases’ inception onwards). Eligibility criteria will include individuals described as having MUD or PMU, with designs of interest including randomized trials, non-randomized trials, and controlled cohort studies with three or more months of follow-up; systematic reviews and CPGs will also be sought. Two reviewers (with support from automation tools) will independently screen all citations, full-text articles, and chart data. Different approaches to handling and summarizing the data will be implemented for each type of study design. Tables and graphics will be used to map evidence sources and identify evidence gaps.

**Discussion:**

This research will enhance awareness of evidence addressing the effects of psychosocial and pharmacologic interventions for MUD/PMU overall and in sub-populations, both in terms of recent CPGs/reviews and primary studies; inspection of the latter will also help establish the feasibility of future syntheses to compare treatments, such as network meta-analysis.

**Systematic review protocol registration:**

Open Science Framework (https://osf.io/9wy8p)

## Background

Methamphetamine use and related harms are rising at an alarming rate in Canada. While data from 2017 suggest that 0.2% of Canadians aged 15 years and older and 3.7% of Canadians from grades 7 to 12 had used methamphetamines within the past 12 months [1], recent data suggest large increases in the numbers of related emergency department visits, hospitalizations, and overdose deaths [1–3]. Between 2010 and 2017, law enforcement agencies in Canadian jurisdictions have identified a 590% increase in the rate of possession and seizure of methamphetamines [1]. Use amongst individuals accessing harm reduction services has also increased [1]. Collectively, these data suggest a growing public health crisis [1, 4, 5]. Social determinants of health drive the methamphetamine problem, including (but not limited to) adverse childhood experiences and trauma, comorbid mental health conditions, and poverty. Methamphetamine also can be used as a “party drug” or as a sexual stimulant given the feelings of wakefulness, euphoria, and sexual arousal it provides [1, 6]. Methamphetamine has a prolonged duration of action with a large percentage of the drug remaining unchanged in the body, therefore remaining in the brain longer and leading to prolonged stimulant effects [7]. Regular consumption is associated with many negative health consequences including a significant rise in the risk of adverse mental health symptoms (e.g., psychosis, paranoia, depression, insomnia, and cognitive impairment [8–11]), increased likelihood of risky behaviors (e.g., illicit drug consumption, criminal behaviors, and sexual behaviors with increased risk of sexually transmitted infections [12–19]), and other physiologic effects (e.g., “meth mouth” [20, 21]; brain abnormalities such as reductions in white-matter integrity and hippocampal volumes, cardiovascular problems, hyperthermia, seizures [6, 22–24]), infections (e.g., human immunodeficiency virus, hepatitis, methicillin-resistant *Staphylococcus aureus*), endocarditis [25, 26], and risk of death from overdose [1, 27].

Methamphetamine use disorder (MUD) is defined as “a pattern of amphetamine-type substance use leading to clinically significant impairment or distress” as manifested by at least two of 11 DSM-5 criteria during a 12-month time frame, including consumption of a larger amount or over a longer period of time than intended, cravings, tolerance, and withdrawal (Additional file 1 provides a full listing of all criteria) [28]. Problematic methamphetamine use (PMU) is less well defined and is a term used in the literature without a focused definition. In addition to the inclusion of studies of substance use disorder or dependency, previous systematic reviews have also considered studies of problematic substance use where participants do not have a formal diagnosis of substance use disorder but meet a particular threshold of use (e.g., daily, monthly), are treatment-seeking for their substance use, or are identified as a result of their use (e.g., substance use-related emergency department visit) [29, 30].

Treatment of individuals with MUD/PMU remains a challenge, with no clear best approach. Treatment efforts and research include psychosocial approaches (e.g., cognitive behavioral therapy, motivational interviewing, and motivational enhancement therapy), as well as pharmacologics that have been explored in smaller studies (e.g., dopamine agonists, GABA agents, antipsychotics, and antidepressants [31–33]) and considered in recent reviews [34]. In addition to addressing the lack of consensus for effective therapy for MUD/PMU in general, there is also a need to establish whether nuances in treatment are needed amongst key subgroups including those with mental health comorbidities (given many people who use methamphetamines have co-existing conditions and methamphetamine use can further exacerbate their challenges [35, 36]); gay, bisexual, and other men who have sex with men, both cisgender and transgender (gbMSM; given the higher prevalence of crystal meth use, use particularly related to sexuality and injection practices in sexual contexts [37], and some evidence-tailored programs may be beneficial [38–40]); adolescents (since earlier exposure and life experiences may have effects not yet well understood [41, 42]); individuals in correction services (because many people are incarcerated as a result of their methamphetamine use [43] and methamphetamine use is associated with an increased likelihood to reoffend and be returned to custody upon release [44]); and pregnant women (e.g., the prevalence amongst pregnant women has tripled in the USA between 1994 and 2006 in terms of admissions to treatment centers [45], suggesting they require special consideration).

The availability of clinical practice guidelines (CPGs) for the treatment of MUD/PMU is extremely limited [46–49]. Development of CPGs relies upon the availability of rigorous systematic reviews and meta-analyses (SR/MA), which are a long-standing cornerstone of evidence-based medicine [50–52]. While a small number of SR/MA evaluating different aspects of treatment from MUD/PMU have been published in the past 20 years [31–34, 53–58], these works suffer from limitations including the grouping of all forms of stimulant and amphetamine use disorders together, which may mask important differences in treatment outcomes associated with different modes of therapy between types of stimulant disorders; failure to consider both psychosocial and pharmacologic treatment alternatives; limited search strategies (associated with risk of missing important literature); and the a priori exclusion of studies focused upon individuals from important clinical subgroups (including those with mental health comorbidities, adolescents, pregnant women, gbMSM, those in the corrections system). Given the lack of CPGs and the limitations of past SR/MAs that can inform future CPG initiatives, there is a need to develop focused syntheses comparing interventions for MUD/PMU and to establish evidence for nuances of treatment in key subpopulations.

## Objectives of this research

In circumstances where the breadth and dimensions of a large body of evidence are not well known and the ability to develop rigorous SR/MAs and CPGs is uncertain, scoping reviews [59, 60] represent an appropriate investigative approach to knowledge synthesis to gain insights regarding the feasibility of these objectives. Scoping reviews differ in purpose from systematic reviews and can be undertaken to (1) examine the extent, range, and nature of research activity on a topic of interest; (2) determine the feasibility and value of undertaking full de novo systematic reviews on specific topics; (3) summarize and disseminate research findings; and (4) identify research gaps in the existing literature. Simultaneously, gaps in the current literature (including understudied interventions, understudied patient populations, and methodologic deficiencies in past studies that must be addressed in future study designs) can be prioritized.

In the planned scoping review, the following research questions will be addressed:

1. *What are the recommendations of available clinical practice guidelines that address psychosocial and pharmacologic treatment of MUD/PMU?*
2. *What are the key characteristics (e.g., types of patients enrolled, treatments compared, outcomes assessed) of available studies evaluating the clinical benefits of psychosocial and pharmacologic interventions for MUD/PMU?* This will establish a comprehensive repository of all available evidence and a map of characteristics across studies (i.e., SRs and primary studies). The results from this mapping exercise will allow for a succinct summation of evidence and will also inform a feasibility evaluation (e.g., judging homogeneity, availability of common outcomes, and patterns of treatment comparisons made) to establish and plan for subsequent advanced evidence syntheses (e.g., meta-analysis and network meta-analyses) that could inform CPG development.
3. *What are the clinical benefits and harms of psychosocial and pharmacological interventions for MUD/PMU?*
4. *What topics require future research to enhance treatment options for MUD/PMU?* Based on the results from research questions 1–3, evidence gaps, and/or areas of prioritization may be identified. This information will be used to work with knowledge users to establish priorities for future research (i.e., a systematic review in the absence of existing high-quality and up-to-date reviews, a network meta-analysis, and/or a clinical practice guideline).

## Methods

This protocol was drafted in accordance with the Preferred Reporting Items for Systematic Reviews and Meta-Analysis Protocols (PRISMA-P) checklist [61] (Additional file 2) and is registered on Open Science Framework (https://osf.io/9wy8p). The scoping review will be conducted using best practices for scoping reviews outlined by Arksey & O’Malley [59], the Joanna Briggs Institute [62], and others [63–65]. Findings from the review will be reported according to guidance from the PRISMA Extension Statement for Scoping Reviews [66]. Amendments to this protocol will be noted in the final report.

### Eligibility criteria

Studies meeting the following eligibility criteria will be included:

### Population

- ▪ Individuals with methamphetamine use disorder [(defined by DSM-5 (Additional file 1)], or methamphetamine dependence, abuse, or addiction as defined by the earlier editions of the DSM, 10th version of the International Classification of Disease (ICD-10) (https://www.icd10data.com/ICD10CM/Codes/F01-F99/F10-F19/F15-/F15.10), or earlier ICD versions;
- ▪ Individuals with PMU whereby participants do not have a formal diagnosis of MUD but are described to be using methamphetamines nearly daily and/or are seeking treatment for methamphetamine use (as has been considered in past reviews of substance use disorder [29]);
- ▪ No restrictions for age or other population characteristics will be used.

### Concept

- ▪ Studies that measure the impact of different psychosocial and pharmacologic interventions for MUD/PMU in terms of changes in methamphetamine (and other substance) use (e.g., abstinence, reduction), study retention/dropout, treatment retention/discontinuation, acceptability of intervention, mental (e.g., depression, quality of life) and physical (e.g., sexually transmitted infections, risk behaviors, hospitalization) health, self-efficacy, withdrawal symptoms (e.g., cravings), legal/employment outcomes, and harms (e.g., morbidity, mortality, adverse events (AE), study withdrawal due to AEs).
  - ○ *Psychosocial interventions* will include (but will not be limited to) individual-based, group-based, and family-based interventions of therapies applied for MUD/PMU, such as cognitive behavioral therapy (CBT) [67], motivational enhancement therapy (MET) [68], contingency management (CM) [69], motivational interviewing (MI) [68], community reinforcement approach (CRA) [69], support group programs, acceptance and commitment therapy [70], dialectical behavioral therapy [71], mindfulness therapy [72], and combinations of these strategies (see Additional file 3 for additional information about these interventions). Studies will be required to have involved control interventions such as drug counseling (DC), treatment as usual (TAU), or another form of psychosocial intervention (to allow for consideration of a future NMA involving *direct* and *indirect* evidence [73–75]).
  - ○ *Pharmacologic interventions* will include (but not be limited to) dopamine agonists [76–80] (e.g., amantadine), psychostimulants [33, 81, 82] (e.g., dextroamphetamine, methylphenidate), gamma-aminobutyric acid agents (GABA; e.g., baclofen, acamprosate, gabapentin), antipsychotics (e.g., aripiprazole [83]), opioid antagonists (e.g., naltrexone [84, 85]), antidepressants [6, 31, 86] (e.g., sertraline, bupropion, mirtazapine, imipramine), cognitive enhancers [31, 32, 87] (e.g., rivastigmine, galantamine, varenicline, modafinil), topiramate [88], and oxytocin [89]. Vaccine interventions will also be of interest [90, 91], and a control group (placebo, no treatment or another active therapy) will be required for all studies.
- ▪ For both types of interventions, we will seek to identify whether evidence of benefits and harms is presented.

### Context

- ▪ Amongst the target population of individuals with MUD/PMU, there will also be interest in data pertaining to key subgroups including individuals with mental health comorbidities; youth; cisgender gbMSM, transgender gbMSM; those with other substance use problems; pregnant women; and individuals in correction services. Data for these and other clinically important subgroups that emerge during the review will be mapped given the potential for differential benefits of different forms of interventions [35, 36, 38–41, 45, 92, 93].
- ▪ Study designs of interest include randomized trials, non-randomized trials, and comparative cohort studies with 3 or more months of follow-up; systematic reviews and CPGs from the past 5 years will also be sought. Case-control studies, mixed-methods studies, qualitative studies, cross-sectional studies, interrupted time series, before-after studies, editorials, letters, commentaries, case series, and case reports will be excluded, as these are unlikely to change decisions regarding the need for future research and will not be included as eligible designs in intended future evidence syntheses for CPG development.
- ▪ There will be no restrictions on setting or geographic location.

### Data sources and search for studies

A draft search strategy has been developed (Additional file 4) and will be further refined through an iterative process by an experienced medical information specialist with input from the study team. Search strategies will be created using appropriate study design filters. The final search strategies will be peer-reviewed using the Peer-Review of Electronic Search Strategies (PRESS) guideline [94]. Using the OVID platform, we will search the following databases (from inception onward): Ovid MEDLINE® ALL, Embase Classic + Embase, PsycINFO, and EBM Reviews databases (Cochrane Database of Systematic Reviews, Cochrane CENTRAL Register of Controlled Trials, Database of Reviews of Effects, and Health Technology Assessment). Strategies will utilize a combination of controlled vocabulary (e.g., “Amphetamine-Related Disorders”, “Methamphetamine”) and keywords (e.g., crystal meth, MUD, PMU); both vocabulary and the search syntax will be adjusted across databases as needed. The search for CPGs and SRs will be limited to the last 5 years (i.e., 2015–2020), as we are identifying the current state of the evidence and older recommendations may no longer be relevant. There will be no date restrictions for the primary study search. There will be no language restriction in any of the searches; however, only English and French language publications will be included, for feasibility (further described below). When possible, conference abstracts, animal-only records, and opinion pieces will be removed from the results. To supplement published peer-reviewed research, a gray literature search will be performed using CADTH’s Grey Matters checklist [95]. This search will be carried out over a maximum of 5 days (approximately 35 h); potentially relevant sources will be brought forward for screening using methods analogous to those for citations identified by the databases search (further described below). Search efforts will be supplemented by contacting authors in the field to identify additional relevant material and scanning the reference lists of relevant CPGs, SR, and included primary studies. The final search results from databases and gray literature searches will be imported to EndNote and duplicates will be removed. Deduplicated results will be imported into DistillerSR (Evidence Partners Inc.), a systematic review managing software, for subsequent study selection.

### Study selection

Study selection will be conducted separately for SRs/CPGs and primary studies using a two-stage screening process. In stage 1, all records identified by the search for SRs/CPGs will be evaluated based on title and abstract. A calibration exercise of 100 records will be performed among all reviewers (independently, in duplicate), with conflicts resolved through discussion. Once conflicts are resolved, the remaining records will be screened using the liberal accelerated method wherein one reviewer is needed to deem a record relevant and two reviewers are needed to exclude it [96].

Given the large yield of records (approximately 13,000) identified by the search for primary studies, we will leverage artificial intelligence (AI) to expedite title and abstract screening. Specifically, the AI prioritization tool built in DistillerSR will be used to prioritize the order in which citations are screened, allowing for faster screening of the most relevant citations. This will allow for subsequent stages of the review to commence sooner (e.g., full-text screening, data extraction) while the remaining less relevant citations are screened concurrently. First, to pilot the screening question and to train the AI reviewer tool in DistillerSR, each member of the review team will independently screen 200 records. All conflicts will be resolved. This training set will be comprised of 10 to15 potentially relevant studies, identified from existing reviews and from clinical experts, to build a more informed training set. Once the AI reviewer is trained (*n* = 200, as determined by 2% of all records, with a minimum of 25 and a maximum of 200 records), remaining citations will be screened by the review team using the liberal accelerated method [96]. As the AI tool uses all information from each previous iteration of 200 records (through active learning), to increase the prioritization accuracy, disagreements between reviewers will be resolved at the end of each day. Once 95% of predicted relevant references have been identified, the remaining records will be excluded by the AI reviewer; these excluded records will be double-checked by a review team member to conform to the liberal accelerated screening method.

In stage 2 screening, the full texts of all records deemed potentially relevant during the review of titles/abstracts will be evaluated against the target population, content, and context criteria outlined earlier. A draft of full-text screening form will be developed by members of the research team, and a calibration exercise of 25 records will be performed (independently, in duplicate), with conflicts resolved through discussion. After this exercise, the screening form will be finalized based on feedback from the team and each remaining record will be evaluated by two independent reviewers. Conflicts will be resolved through discussion, or a third team member, when required. Reports that are co-publications or multiple reports of the same study will be identified and labeled as such. For full-text screening, where study eligibility is unclear due to limitations in reporting, the corresponding author will be contacted by email twice, 2 weeks apart, for additional information. If no response is received, the article will be excluded and will be included in the list of excluded studies as “unclear” for the related question in the screening form. Only studies published in English or French (for reasons of timeliness and costs) will be included. A list of potentially relevant publications in other languages will be made available. Once study selection is completed, a PRISMA flow diagram documenting the process will be prepared for inclusion in the final report [97].

Using Robinson et al. [98] and Methodological Expectations of Cochrane Intervention Reviews (MECIR) [99] as guidance, SRs will be required to meet the following criteria for inclusion (otherwise, they will be considered a narrative review): (i) at least two databases were searched, (ii) it reports selection criteria, (iii) quality appraisal of included studies is reported, and (iv) it provides a list and synthesis of included studies. Additionally, CPGs will be included only if (i) the guideline development panel was comprised of diverse and relevant stakeholders, such as health professionals, methodologists, and experts on the topic; (ii) the process used to reach consensus among panel members and the methods for guideline development was transparently described; (iii) the guideline disclosed its funding and the conflicts of interest (financial and non-financial) for members of the guideline development panel; (iv) systematic review methods were used to identify and evaluate evidence; and (v) guideline recommendations were clearly stated and, for each recommendation, there was a clear description of potential benefits and harms provided in a summary of the available evidence with a rating of the level of confidence or strength of the evidence underpinning each recommendation provided.

### Data extraction

A meeting will be held with the research team to finalize a data-charting form to determine the variables to be extracted (e.g., study characteristics, population demographics, interventions, outcomes). The data-charting form will be implemented in DistillerSR. A draft list of variables to be extracted for each study type of interest is provided in Additional file 5. From primary studies such as randomized controlled trials, we will collect elements related to study design methodology, interventions compared, participant enrollment criteria, participants’ baseline demographic measures, outcomes reported (amongst those of interest described earlier), and key clinical interpretations drawn. Review eligibility criteria, search dates covered, a number of included studies and key findings will be collected from systematic reviews. For clinical practice guidelines, the year of issue, methods of development and recommendations (with corresponding cited strength of evidence, if available) will be extracted. For each study type, a calibration exercise of five studies will be performed by all reviewers, and feedback regarding content will be incorporated into the data-charting form before proceeding with the charting of the remaining literature. One reviewer will chart the data, and a second reviewer will verify all data. Adjustments may be made to the data-charting form in an iterative process, as necessary [59, 62]. Any disagreements will be resolved through discussion or adjudication by a third reviewer, if required. In cases where outcome data are not clearly reported, the corresponding author will be contacted for clarification. If no response is received, the article will be included, but data will not be provided for that outcome.

### Quality appraisal of the evidence

As this scoping review is being conducted to provide an overview of existing evidence and gaps (regardless of methodological quality), the risk of bias assessments of primary research studies will not be undertaken. However, as SR/MAs and CPGs will also be used to identify gaps and prioritization for future research needs (through a steering committee consisting of membership from researchers and knowledge users), we will evaluate the quality of conduct of these documents. SR/MAs will be evaluated using the AMSTAR 2 checklist [100], while CPGs will be evaluated using the AGREE-2 checklist [101].

### Synthesis/charting of data and feasibility evaluation for future meta-analyses

Different approaches to charting will be implemented for each type of study design. The approaches to be used are outlined below, and supplements will be used in the review as necessary to present all relevant information for readers.

- *CPGs:* The chronologic age, approach to development (including support from SR/MA, the involvement of content experts and participants with lived experience, the use of grading methods such as GRADE [102]), and the nature of recommendations (and grades) made will be summarized; AGREE-2 evaluations [101] will be summarized to give insights as to the rigor of CPG development.
- *SR/MAs:* The chronologic age (e.g., date published, date of search), nature of SR/MA methods, treatment comparisons made, and key findings in included SR/MAs will be collected and summarized; AMSTAR-2 evaluations [100] will provide an assessment of their internal validity. This will allow for identification and examination of the quality of existing reviews and determine if an updated review should be performed.
- *Primary studies of interventions:* Primary studies will be grouped first by outcome (e.g., abstinence, reduction, treatment retention/discontinuation, changes in mental health), then by study design (e.g., RCT, cohort study), and intervention type (e.g., psychosocial, pharmacologic, combined). Study characteristics (e.g., year, country, number of participants and demographics, outcomes, context, and details regarding methamphetamine use) will be presented in tabular form, with trends in key factors being narratively summarized within the final report. Studies with available data for subgroups of interest will be mapped to assess the feasibility of future syntheses and to note whether the identified literature identified important considerations for treatment. Results will be summarized by population and design for each effect and presented using tables and visuals [e.g., outcome maps, bar charts, bubble plots], as deemed most interpretable by the research team and dependent upon the quantity of information to be summarized. Per outcome, evidence network diagrams will be prepared to visualize the available evidence base and to consider the potential for NMAs in the context of a full systematic review. Per outcome, median values and ranges of treatment effects for each treatment comparison will be described. A narrative summary will be used to further describe the results from the tables and charts. Organizing data first by outcome will allow identification of (1) comparisons across study design types, (2) contradictory results (e.g., findings from different studies which suggest contrary results related to abstinence from methamphetamines, retention in treatment), and (3) gaps in the evidence for each effect. This will also allow for recognition of which effects have not been evaluated or where evaluation is sparse. It will also identify if an NMA can or should be performed and will highlight covariates which may be considered for exploration using subgroup analyses or meta-regression.

## Discussion

Several past reviews of treatments for MUD/PMU have excluded special populations (e.g., pregnant women), did not include comparative cohort studies, and have grouped all forms of stimulant and amphetamine use disorders together. The current review will seek to address these limitations by means of a focused synthesis of treatments specifically for MUD/PMU as well as by incorporating evidence for special populations in order to establish evidence for nuances of treatment. We anticipate certain challenges during study selection with regard to studies involving the treatment of MUD/PMU and other conditions concurrently, and we will work closely with our clinical experts to ensure all relevant evidence is included.

In addition to disseminating findings via publication and through the networks of our knowledge users, we will establish a steering committee, consisting of content experts, methodologists, Canadian agency stakeholders/decision-makers (including representation from the Canadian Center on Substance Use and Addiction, the Canadian Society of Addiction Medicine, Public Health Ontario, Correctional Service Canada, and other organizations), and people with lived experience (including representation from the Community Addictions Peer Support Association, the Drug Users’ Advocacy League and Max Ottawa), for engagement in a prioritization exercise based upon our findings; these groups will participate throughout this research initiative. The prioritization exercise will use an approach that will consist of (i) a webinar to present and discuss findings from the scoping review to be held 1 week after sharing of the final report, (ii) a Delphi survey of team members and knowledge users to establish preliminary perspectives on research priorities based on the study findings that were presented, and (iii) a second webinar of all contributors to discuss results from the survey, with the objective of informing a ranking of priorities with input from all parties. We anticipate the second component to focus upon the need and feasibility for new SR/MAs and CPGs for interventions to treat MUD/PMU, as well as aspects of research of interventions to manage MUD/PMU where future primary research is needed (which may include interventions in apparent need of further study, key subgroups of individuals with MUD/PMU where more research is needed, and possibly other methodologic issues such as the need for core outcome sets to guide future trials) [103, 104]. A listing of the final priorities will be shared with the knowledge users for dissemination to inform and influence policy makers and health priorities and will also be shared with our funder to be considered toward the establishment of future funding opportunities.

## Supplementary information

**Additional file 1.** DSM-5 criteria for Methamphetamine Use Disorder

**Additional file 2.** PRISMA-P checklist.

**Additional file 3.** Definitions of Psychosocial Interventions and Comparators for MUD/PMU.

**Additional file 4.** Draft Medline Literature Search.

**Additional file 5.** Draft list of data charting components.

## Data Availability

All data and materials associated with this protocol are available. All data and materials for the completed scoping review will be made available at the time of publication.

## Abbreviations

AE: Adverse event
CBT: Cognitive behavioral therapy
CM: Contingency management
CPG: Clinical practice guideline
CRA: Community reinforcement approach
gbMSM: Gay, bisexual, and other men who have sex with men
MET: Motivational enhancement therapy
MI: Motivational interviewing
MUD: Methamphetamine use disorder
NMA: Network meta-analysis
PMU: Problematic methamphetamine use
PRISMA: Preferred Reporting Items for Systematic Reviews and Meta-Analyses
RCT: Randomized controlled trial
SR/MA: Systematic review and meta-analysis
