# Supplementary material for: Psychosocial and pharmacologic interventions for methamphetamine addiction: protocol for a scoping review of the literature
Source: Syst Rev. 2020 Oct 24;9:245. doi: 10.1186/s13643-020-01499-z (PMC7585172; doi:10.1186/s13643-020-01499-z)
Supplement: Supplementary file 1 — Additional file 1. DSM-5 criteria for Methamphetamine Use Disorder [file 13643_2020_1499_MOESM1_ESM.docx]

## Appendix 1. DSM-5 Criteria for Methamphetamine Use Disorder

According to the **DSM-5**, there is ***one*** main methamphetamine-related mental health disorder: a stimulant use disorder. This is defined as follows:

A pattern of amphetamine-type substance, cocaine, or other stimulant use leading to clinically significant impairment or distress, as manifested by at least two of the following, occurring within a 12-month period:

1. The stimulant is often taken in larger amounts or over a longer period than was intended
2. There is a persistent desire or unsuccessful efforts to cut down or control stimulant use
3. A great deal of time is spent in activities necessary to obtain the stimulant, use the stimulant, or recover from its effects
4. Craving, or a strong desire or urge to use the stimulant
5. Recurrent stimulant use resulting in a failure to fulfil major role obligations at work, school, or home
6. Continued stimulant use despite having persistent or recurrent social or interpersonal problems caused or exacerbated by the effects of the stimulant
7. Important social, occupational, or recreational activities are given up or reduced because of stimulant use
8. Recurrent stimulant use in situations in which it is physically hazardous
9. Stimulant use is continued despite knowledge of having a persistent or recurrent physical or psychological problem that is likely to have been caused or exacerbated by the stimulant
10. Tolerance, as defined by either of the following:
    1. A need for markedly increased amounts of the stimulant to achieve intoxication or desired effect.
    2. A markedly diminished effect with continued use of the same amount of the stimulant.
       **Note:** This criterion is not considered to be met for those taking stimulant medications solely under appropriate medical supervision, such as medications for attention-deficit/hyperactivity disorder or narcolepsy.
11. Withdrawal, as manifested by either of the following:
    1. The characteristic withdrawal syndrome for the stimulant (refer to Criteria A and B of the criteria set for stimulant withdrawal).^[[1]](#footnote-1)^
    2. The stimulant (or a closely related substance) is taken to relieve or avoid withdrawal symptoms.
       **Note:** This criterion is not considered to be met for those taking stimulant medications solely under appropriate medical supervision, such as medications for attention-deficit/hyperactivity disorder or narcolepsy.

A ***mild*** stimulant use disorder is defined as the presence of 2-3 of the above symptoms.

A ***moderate*** stimulant use disorder is defined as the presence of 4-5 of the above symptoms.

A ***severe***stimulant use disorder is defined as the presence of 6 or more of the above symptoms.

1. American Psychiatric Association. Diagnostic and Statistical Manual of Mental Disorders. 5th ed. Washington, DC: American Psychiatric Publishing; 2013. [↑](#footnote-ref-1)
