# Supplementary material for: Psychosocial and pharmacologic interventions for methamphetamine addiction: protocol for a scoping review of the literature
Source: Syst Rev. 2020 Oct 24;9:245. doi: 10.1186/s13643-020-01499-z (PMC7585172; doi:10.1186/s13643-020-01499-z)
Supplement: Supplementary file 3 — Additional file 3. Definitions of Psychosocial Interventions and Comparators for MUD/PMU. [file 13643_2020_1499_MOESM3_ESM.docx]

## Appendix 3. Definitions of Psychosocial Interventions and Comparators for MUD/PMU

Provided below are descriptions of the a priori list of psychosocial interventions for which we anticipate finding study data and which are considered of clinical relevance for the planned knowledge syntheses. Other interventions may also be encountered during the review and will be discussed by the research team for relevance as they are encountered. When needed, psychosocial interventions which are not labeled using a known terminology or which are different in administration than the core descriptions of these interventions will also be reviewed for relevance by clinical members of the research team.

**Active Control Groups**

- Delayed treatment, case management and drug counseling

**Psychosocial Interventions for MUD/PMU**

*Individual Interventions*

1. ***Acceptance and Commitment Therapy (ACT)***: this therapy views psychological events as ongoing actions of the whole organism interacting in and with historically and situationally defined contexts. From an ACT perspective, patients devote most of their time and energy to flee from personal and uncomfortable experiences. This keeps them from being engaged in life activities. ACT sessions may include: assessing values and considering how methamphetamine use may interfere with these values, creative hopelessness related to the effectiveness of methamphetamine use, exploring discrepancies between control strategies and values, accepting rather than engaging in urges to use methamphetamine, and discussing action plans (1).
2. ***Cognitive behavioral therapy (CBT)***: this therapy is a short-term, present-focused therapy oriented towards helping people adjust unhelpful thinking patterns in order to change behaviour and improve emotions. The main components of this counseling focus on performing a functional analysis of behavior, promoting behavioral activation, identifying and coping with methamphetamine cravings, enhancing drug-refusal skills, enhancing decision-making about high-risk situations, and improving problem-solving skills (2).
3. ***Contingency Management (CM) (i.e., medication, prize, or monetary equivalent reinforcement)***: this behavioral treatment is based on positive/negative reinforcers used to promote abstinence from methamphetamine use during treatment. With positive reinforcers, the participants can obtain payment for methamphetamine-free urine (3).
4. ***Community Reinforcement Approach***: (CRA) this behavioural approach aims to promote abstinence of methamphetamines. During sessions, participants are provided with relationship and employment counselling, instructions on antecedents and consequences of their methamphetamine use, assistance in developing new or reinitiating old recreational activities, and are connected to other community resources if needed. Significant others are also often mobilized to administer reinforcers contingent on non-methamphetamine using behavior of the patient (3).
5. ***Dialectical Behavior Therapy (DBT)***: applies directive, problem-oriented techniques that are balanced with supportive techniques, such as reflection, empathy, acceptance and emphasis on the client’s inherent ability to access an internal ‘wise mind’. In addition, dialectical strategies are employed, including balancing acceptance with change, alternating validation with problem solving and using paradox and metaphor to reduce methamphetamine use. Individual DBT targets maladaptive behaviors in hierarchical order (suicidal, therapy-interfering, substance use, and quality-of-life interfering behaviors) and replaces those behaviors with skillful behaviors learned in a psycho-educational skills group (4).
6. ***Mindfulness-Based Therapy***: Individually delivered mindfulness techniques. “Mindfulness is a technique comprised of meditation and a specific mental tendency toward experience that encourages an awareness of the present-moment.” Sessions included: meditation techniques, identifying triggers and cravings for methamphetamine use through mindfulness, mindfulness in high risk situations, acceptance and skillful action, seeing thoughts as thoughts, self-care, and social support practices (5). Mindfulness-based therapy can also include specialized focus on relapse prevention (6).
7. ***Motivational Enhancement Therapy (MET)****:* this counseling approach helps individuals resolve their ambivalence about engaging in treatment and stopping their methamphetamine use. This intervention seeks to bring about rapid and internally motivated change, rather than guiding participants stepwise through the recovery process.
8. ***Motivational Interviewing (MI)*:** this approach is a non-confrontational, goal-oriented process designed to improve readiness for behavior change and help clients resolve ambivalence towards reducing methamphetamine use (7).

***Couples Interventions for MUD/PMU***

1. ***Behavioral Couples Therapy****:* considers the role methamphetamine use and relationship concerns. Treatment is skills based and emphasizes partners’ specification (objectification) of desired positive changes from their partner, with various reinforcements of desired behavior change (i.e., reducing methamphetamine use), leading to behavioral exchanges.” (8)
2. ***Cognitive-Behavioral Couples Therapy****:* time-limited therapy that involves the couple. Therapy is relatively structured and involves goal setting. A functional analysis is often done to assess maladaptive affective and instrumental behaviors and the environmental contingencies supporting methamphetamine use, and the ways in which the couple’s reciprocal interactions affect their relational satisfaction. Based on the functional analysis, the therapist and couple together delineate specific treatment goals which often involve increasing exchanges of positive behaviours and improving communication skills that influence the interaction process within the couple in order to reduce methamphetamine use (8).
3. ***Emotion- Focused Couples Therapy****:* adheres to humanistic– experiential therapy principles: 1. The therapeutic alliance can itself be healing; 2. the inherent validity of the patient’s experience is central to change of methamphetamine use, and is fostered by the therapist’s authenticity and transparency; 3. given the opportunity to do so, people have the ability to make healthy choices; 4. both the inner and outer realities of people’s lives need to be attended to; and 5. therapy can provide opportunities for direct, in- session, corrective emotional experiences to facilitate reductions in methamphetamine use (8).

***Family Interventions for MUD/PMU***

1. ***Behavioral Family Therapy***: “based on social learning theory and behavior exchange principles derived from classical and operant conditioning approaches.” This therapy aims to improve interactions between family members through reinforcement of desired behavior change, specifically, the reduction of methamphetamine use (8).
2. ***Cognitive-Behavioral Family Therapy****:* time-limited therapy that involves the whole family, or subgroups of the family. Therapy is relatively structured and involves goal setting. A functional analysis is often done to assess maladaptive affective and instrumental behaviors and the environmental contingencies maintaining methamphetamine use, and the ways in which family members’ reciprocal interactions affect their relational satisfaction. Based on the functional analysis, the therapist and family together delineate specific treatment goals which often involve reducing methamphetamine use, increasing exchanges of positive behaviours and improving communication skills that influence the interaction process within the family (8).
3. ***Emotion- Focused Family Therapy****:* Family delivered format of emotion focused therapy. Adheres to humanistic– experiential therapy principles: 1. The therapeutic alliance can itself be healing; 2. the inherent validity of the patient’s experience is central to change of methamphetamine use, and is fostered by the therapist’s authenticity and transparency; 3. given the opportunity to do so, people have the ability to make healthy choices; 4. both the inner and outer realities of people’s lives need to be attended to; and 5. therapy can provide opportunities for direct, in- session, corrective emotional experiences to facilitate reductions in methamphetamine use (8).
4. ***Multi-Dimensional Family Therapy****:* an empirically supported, manualized, family intervention that can be applied for adolescent methamphetamine use. It aims to change, among other things, parenting behaviors and family interactions in order to help reduce methamphetamine use. It typically also involves a focus on the adolescent individually (e.g., self-efficacy and social skills) and targets known determinants of adolescent substance use (9).

***Group Interventions for MUD/PMU***

1. ***12-Step Facilitation Group***: Amphetamines Anonymous meetings are part of a self-help, peer-group that supports people who use drugs. Amphetamines anonymous is structured from the basic principles of alcoholics anonymous (AA) groups, where members are encouraged to learn from one-another to live a methamphetamine-free life.
2. ***Acceptance and Commitment Therapy***: Group delivered format of acceptance and commitment therapy. This therapy views psychological events as ongoing actions of the whole organism interacting in and with historically and situationally defined contexts. From an ACT perspective, patients devote most of their time and energy to flee from personal and uncomfortable experiences. This keeps them from being engaged in life activities. ACT sessions may include: assessing values and considering how methamphetamine use may interfere with these values, creative hopelessness related to the effectiveness of methamphetamine use, exploring discrepancies between control strategies and values, accepting rather than engaging in urges to use methamphetamine, and discussing action plans (1).
3. ***Cognitive Behavioral Group Therapy***: Group delivered format of cognitive behavioral therapy. This therapy is a short-term, present-focused therapy oriented towards helping people adjust unhelpful thinking patterns in order to change behaviour and improve emotions. The main components of this counseling focus on performing a functional analysis of behavior, promoting behavioral activation, identifying and coping with methamphetamine cravings, enhancing drug-refusal skills, enhancing decision-making about high-risk situations, and improving problem-solving skills.
4. ***Dialectical Behavior Group Therapy***: Group delivered format of dialectical behavior therapy. This therapy applies directive, problem-oriented techniques that are balanced with supportive techniques, such as reflection, empathy, acceptance and emphasis on the client’s inherent ability to access an internal ‘wise mind’. In addition, dialectical strategies are employed, including balancing acceptance with change, alternating validation with problem solving and using paradox and metaphor to reduce methamphetamine use. Individual DBT targets maladaptive behaviors in hierarchical order (suicidal, therapy-interfering, substance use, and quality-of-life interfering behaviors) and replaces those behaviors with skillful behaviors learned in a psycho-educational skills group (4).
5. ***Group Motivational Enhancement Therapy:*** Group delivered motivational enhancement therapy. This counseling approach helps individuals resolve their ambivalence about engaging in treatment and stopping their methamphetamine use. This intervention seeks to bring about rapid and internally motivated change, rather than guiding participants stepwise through the recovery process.
6. ***Group Motivational Interviewing***: Group delivered format of motivational interviewing. A non-confrontational, goal-oriented process designed to improve readiness for behavior change and help clients resolve ambivalence towards reducing methamphetamine use (7).

***Mindfulness-Based Group Therapy***: Group delivered mindfulness techniques. “Mindfulness is a technique comprised of meditation and a specific mental tendency toward experience that encourages an awareness of the present-moment.” Sessions included: meditation techniques, identifying triggers and cravings for methamphetamine use through mindfulness, mindfulness in high risk situations, acceptance and skillful action, seeing thoughts as thoughts, self-care, and social support practices (5).

**References**

1. Saedy M, Kooshki S, Jamali Firouzabadi M, Emamipour S, Rezaei Ardani A. Effectiveness of Acceptance-Commitment Therapy on Anxiety and Depression among Patients on Methadone Treatment: A Pilot Study. Iran J Psychiatry Behav Sci. 2015 Mar;9(1):e222.

2. Moore BA, Fiellin DA, Cutter CJ, Buono FD, Barry DT, Fiellin LE, et al. Cognitive Behavioral Therapy Improves Treatment Outcomes for Prescription Opioid Users in Primary Care Buprenorphine Treatment. J Subst Abuse Treat. 2016 Dec;71:54–7.

3. Amato L, Minozzi S, Davoli M, Vecchi S. Psychosocial combined with agonist maintenance treatments versus agonist maintenance treatments alone for treatment of opioid dependence. Cochrane Database Syst Rev. 2011 Oct 5;(10):CD004147.

4. Linehan MM, Dimeff LA, Reynolds SK, Comtois KA, Welch SS, Heagerty P, et al. Dialectical behavior therapy versus comprehensive validation therapy plus 12-step for the treatment of opioid dependent women meeting criteria for borderline personality disorder. Drug Alcohol Depend. 2002 Jun 1;67(1):13–26.

5. Imani S, Atef Vahid MK, Gharraee B, Habibi M, Bowen S, Noroozi A. Comparing Mindfulness-Based Group Therapy With Treatment as Usual for Opioid Dependents: A Pilot Randomized Clinical Trial Study Protocol. Iran J Psychiatry Behav Sci. 2015 Mar;9(1):e216.

6. Grant S, Colaiaco B, Motala A, Shanman R, Booth M, Sorbero M, et al. Mindfulness-based Relapse Prevention for Substance Use Disorders: A Systematic Review and Meta-analysis. J Addict Med. 2017 Oct;11(5):386–96.

7. Nyamathi A, Shoptaw S, Cohen A, Greengold B, Nyamathi K, Marfisee M, et al. Effect of motivational interviewing on reduction of alcohol use. Drug Alcohol Depend. 2010 Feb 1;107(1):23–30.

8. Messer SB, Gurman AS. Essential Psychotherapies: Theory and Practice. Guilford Press; 2011.

9. Liddle H. Multidimensional Family Therapy. In: Handbook of Family Therapy (2nd edition). New York, USA: Routledge; 2015.
