# Supplementary material for: Psychosocial and pharmacologic interventions for methamphetamine addiction: protocol for a scoping review of the literature
Source: Syst Rev. 2020 Oct 24;9:245. doi: 10.1186/s13643-020-01499-z (PMC7585172; doi:10.1186/s13643-020-01499-z)
Supplement: Supplementary file 4 — Additional file 4. Draft Medline Literature Search. [file 13643_2020_1499_MOESM4_ESM.docx]

## Appendix 4. Draft Medline Literature Search

*Systematic Reviews/Guidelines*

Database: Embase Classic+Embase <1947 to 2020 April 13>, Ovid MEDLINE(R) ALL <1946 to April 13, 2020>, APA PsycInfo <1806 to April Week 1 2020>, EBM Reviews - Cochrane Database of Systematic Reviews <2005 to April 08, 2020>, EBM Reviews - Database of Abstracts of Reviews of Effects <1st Quarter 2016>, EBM Reviews - Health Technology Assessment <4th Quarter 2016>

Search Strategy:

--------------------------------------------------------------------------------

1 Amphetamine-Related Disorders/ (3387)

2 Cocaine-Related Disorders/ (14214)

3 1 or 2 [AMPHETAMINE/COCAINE-RELATED DISORDERS] (17157)

4 Methamphetamine/ (33144)

5 methamphetamine*.tw,kf,rn. (40639)

6 (adipex or ambar or amphedroxyn or cidrin or corvitin or desoxyephedrine hydrochloride or daropervamin or delfetamine stedytab? or deofed or deoxyephedrine or desamine or desfedrin or desoxo or desoxyephedrine or desoxyfed or desoxyn or destim or desyphed or detrex or dextrim or dextro desoxyephedrine or dextro methylamphetamine or dextrolevo deoxyephedrine or dextromethamphetamine or dexyfed or doe or doxephrin or doxyfed or drinalfa or effroxine or efroxinor efroxine or esophan or estimulex or eufodrin or eufodrinal or gerovit or hiropon or isophan or isophen or kemodrin or madrine or metamfetamine or metamphetamine or methamine or methampex or methamphetamin or methamphetaminium or methamphin or methedrine or methoxyn or methyl amphetamine or methylamphetamine or methylbenzedrine or methylisamin or methylisomyn or methylpropamine or miller drine or methyl amphetamine or methylamphetamine or n-methylamphetamine or neopharmedrine or normadrine or norodin or norodrin or oxydess or oxydrene or oxyfed or pervitin or phenylisopropylmethylamine hydrochloride or phenylmethylaminopropane hydrochloride or philipon or philopon or premodrin or psykoton or semoxydrine or soxysympamine or syndroxor tonedron).tw,kf. (11156)

7 (crystal meth* or crank).tw,kf. (3444)

8 uppers.tw,kf. (172)

9 Central Nervous System Stimulants/ (38260)

10 (central nervous system stimulant? or CNS stimulant?).tw,kf. (2599)

11 (psychostimulant? or psycho-stimulant?).tw,kf. (18445)

12 ((stimulant? or stimulating or stimulative) adj (agent or agents or drug or drugs)).tw,kf. (14897)

13 exp Cocaine/ (96458)

14 cocaine.tw,kf. (107331)

15 or/4-14 [METHAMPHETAMINE/CNS STIMULANTS/COCAINE] (229962)

16 Substance-Related Disorders/ (120873)

17 ((drug? or drug-related) adj2 (abus* or addict* or depend* or habituat* or misus* or mis-us* or problem* or "use disorder" or "use disorders")).tw,kf. (185053)

18 drug-related disorder?.tw,kf. (197)

19 ((substance* or substance-related) adj2 (abus* or addict* or depend* or habituat* or misus* or mis-us* or problem* or "use disorder" or "use disorders")).tw,kf. (163063)

20 substance-related disorder?.tw,kf. (3078)

21 (MUD or MUDs).tw,kf. (14983)

22 (PMU or PMUs).tw,kf. (558)

23 or/16-22 [SUBSTANCE DISORDERS] (411488)

24 15 and 23 [METHAMPHETAMINE DISORDERS/PROBLEMATIC USE] (51607)

25 ((methamphetamine* or adipex or ambar or amphedroxyn or cidrin or corvitin or desoxyephedrine hydrochloride or daropervamin or delfetamine stedytab? or deofed or deoxyephedrine or desamine or desfedrin or desoxo or desoxyephedrine or desoxyfed or desoxyn or destim or desyphed or detrex or dextrim or dextro desoxyephedrine or dextro methylamphetamine or dextrolevo deoxyephedrine or dextromethamphetamine or dexyfed or doe or doxephrin or doxyfed or drinalfa or effroxine or efroxinor efroxine or esophan or estimulex or eufodrin or eufodrinal or gerovit or hiropon or isophan or isophen or kemodrin or madrine or metamfetamine or metamphetamine or methamine or methampex or methamphetamin or methamphetamine or methamphetaminium or methamphin or methedrine or methoxyn or methyl amphetamine or methylamphetamine or methylbenzedrine or methylisamin or methylisomyn or methylpropamine or miller drine or methyl amphetamine or methylamphetamine or n-methylamphetamine or neopharmedrine or normadrine or norodin or norodrin or oxydess or oxydrene or oxyfed or pervitin or phenylisopropylmethylamine hydrochloride or phenylmethylaminopropane hydrochloride or philipon or philopon or premodrin or psykoton or semoxydrine or soxysympamine or syndroxor tonedron) adj2 (abus* or addict* or depend* or habituat* or misus* or mis-us* or problem* or "use disorder" or "use disorders")).tw,kf. (5994)

26 (stimulant? adj2 (abus* or addict* or depend* or habituat* or misus* or mis-us* or problem* or "use disorder" or "use disorders")).tw,kf. (3868)

27 ((psychostimulant? or psycho-stimulant?) adj2 (abus* or addict* or depend* or habituat* or misus* or mis-us* or problem* or "use disorder" or "use disorders")).tw,kf. (2066)

28 ((stimulating or stimulative) adj (agent or agents or drug or drugs) adj2 (abus* or addict* or depend* or habituat* or misus* or mis-us* or problem* or "use disorder" or "use disorders")).tw,kf. (21)

29 (cocaine adj2 (abus* or addict* or depend* or habituat* or misus* or mis-us* or problem* or "use disorder" or "use disorders")).tw,kf. (23812)

30 or/25-29 (34279)

31 3 or 24 or 30 [METHAMPHETAMINE USE DISORDERS/PROBLEMATIC USE] (80005)

32 exp Animals/ not Humans/ (18167309)

33 31 not 32 [ANIMAL-ONLY REMOVED] (54359)

34 (comment or editorial or news or newspaper article).pt. (2052084)

35 (letter not (letter and randomized controlled trial)).pt. (2174788)

36 33 not (34 or 35) [OPINION PIECES REMOVED] (52274)

37 systematic review.pt. (133818)

38 exp systematic reviews as topic/ (27943)

39 meta analysis.pt. (113366)

40 exp meta-analysis as topic/ (60821)

41 (meta-analy* or metanaly* or metaanaly* or met analy* or integrative research or integrative review* or integrative overview* or research integration or research overview* or collaborative review*).tw,kf. (469679)

42 (systematic review* or systematic overview* or evidence-based review* or evidence-based overview* or (evidence adj3 (review* or overview*)) or meta-review* or meta-overview* or meta-synthes* or rapid review* or "review of reviews" or umbrella review? or technology assessment* or HTA or HTAs).tw,kf. (584620)

43 exp Technology assessment, biomedical/ (25678)

44 (cochrane or health technology assessment or evidence report or systematic reviews).jw. (58222)

45 (network adj (MA or MAs)).tw,kf. (28)

46 (NMA or NMAs or MTC or MTCs or MAIC or MAICs).tw,kf. (18135)

47 indirect* compar*.tw,kf. (6557)

48 (indirect treatment* adj1 compar*).tw,kf. (930)

49 (mixed treatment* adj1 compar*).tw,kf. (1503)

50 (multiple treatment* adj1 compar*).tw,kf. (460)

51 (multi-treatment* adj1 compar*).tw,kf. (8)

52 simultaneous* compar*.tw,kf. (2630)

53 mixed comparison?.tw,kf. (143)

54 or/37-53 (971870)

55 36 and 54 [REVIEWS] (1258)

56 Critical Pathways/ (15042)

57 exp Clinical Protocols/ (264261)

58 Consensus/ (81254)

59 exp Consensus Development Conference/ (36062)

60 exp Consensus Development Conferences as Topic/ (27009)

61 exp Guideline/ (33667)

62 Guidelines as Topic/ (410933)

63 Practice Guidelines as Topic/ (422003)

64 Health Planning Guidelines/ (100972)

65 (Guideline or Practice Guideline or Consensus Development Conference or Consensus Development Conference, NIH).pt. (42965)

66 (position statement* or policy statement* or practice parameter* or best practice*).tw,kf. (96490)

67 (standards or guideline or guidelines).ti,kf. (250448)

68 ((practice or treatment* or clinical) adj guideline*).ab. (106289)

69 (CPG or CPGs).ti. (12648)

70 consensus*.ti,kf. (58115)

71 consensus*.ab. /freq=2 (62404)

72 ((critical or clinical or practice) adj2 (path or paths or pathway or pathways or protocol*)).tw,kf. (52645)

73 recommendat*.ti,kf. (95422)

74 (overview? adj2 guideline?).tw,kf. (299)

75 or/56-74 [CPG FILTER] (1429270)

76 36 and 75 [CPGs] (704)

77 55 or 76 [REVIEWS, CPGs] (1904)

78 limit 77 to yr="2015-current" [Limit not valid in DARE; records were retained] (680)

79 78 use medall [MEDLINE RECORDS] (244)

80 methamphetamine dependence/ (1477)

81 methamphetamine-induced psychosis/ (77)

82 cocaine dependence/ (20725)

83 cocaine-induced psychosis/ (14)

84 or/80-83 [AMPHETAMINE/COCAINE-RELATED DISORDERS] (21925)

85 Methamphetamine/ (33144)

86 methamphetamine*.tw,kw,rn. (40776)

87 (adipex or ambar or amphedroxyn or cidrin or corvitin or desoxyephedrine hydrochloride or daropervamin or delfetamine stedytab? or deofed or deoxyephedrine or desamine or desfedrin or desoxo or desoxyephedrine or desoxyfed or desoxyn or destim or desyphed or detrex or dextrim or dextro desoxyephedrine or dextro methylamphetamine or dextrolevo deoxyephedrine or dextromethamphetamine or dexyfed or doe or doxephrin or doxyfed or drinalfa or effroxine or efroxinor efroxine or esophan or estimulex or eufodrin or eufodrinal or gerovit or hiropon or isophan or isophen or kemodrin or madrine or metamfetamine or metamphetamine or methamine or methampex or methamphetamin or methamphetaminium or methamphin or methedrine or methoxyn or methyl amphetamine or methylamphetamine or methylbenzedrine or methylisamin or methylisomyn or methylpropamine or miller drine or methyl amphetamine or methylamphetamine or n-methylamphetamine or neopharmedrine or normadrine or norodin or norodrin or oxydess or oxydrene or oxyfed or pervitin or phenylisopropylmethylamine hydrochloride or phenylmethylaminopropane hydrochloride or philipon or philopon or premodrin or psykoton or semoxydrine or soxysympamine or syndroxor tonedron).tw,kw. (11189)

88 (crystal meth* or crank).tw,kw. (3491)

89 uppers.tw,kw. (172)

90 central stimulant agent/ (19256)

91 psychostimulant agent/ (5795)

92 (central nervous system stimulant? or CNS stimulant?).tw,kw. (2742)

93 (psychostimulant? or psycho-stimulant?).tw,kw. (19091)

94 ((stimulant? or stimulating or stimulative) adj (agent or agents or drug or drugs)).tw,kw. (15024)

95 cocaine/ (94794)

96 cocaine.tw,kw. (108149)

97 or/85-96 [METHAMPHETAMINE/CNS STIMULANTS/COCAINE] (219702)

98 drug dependence/ (151784)

99 ((drug? or drug-related) adj2 (abus* or addict* or depend* or habituat* or misus* or mis-us* or problem* or "use disorder" or "use disorders")).tw,kw. (187283)

100 drug-related disorder?.tw,kw. (201)

101 ((substance* or substance-related) adj2 (abus* or addict* or depend* or habituat* or misus* or mis-us* or problem* or "use disorder" or "use disorders")).tw,kw. (165397)

102 substance-related disorder?.tw,kw. (4094)

103 (MUD or MUDs).tw,kw. (14940)

104 (PMU or PMUs).tw,kw. (560)

105 or/98-104 [SUBSTANCE DISORDERS] (431501)

106 97 and 105 [METHAMPHETAMINE DISORDERS/PROBLEMATIC USE] (55106)

107 ((methamphetamine* or adipex or ambar or amphedroxyn or cidrin or corvitin or desoxyephedrine hydrochloride or daropervamin or delfetamine stedytab? or deofed or deoxyephedrine or desamine or desfedrin or desoxo or desoxyephedrine or desoxyfed or desoxyn or destim or desyphed or detrex or dextrim or dextro desoxyephedrine or dextro methylamphetamine or dextrolevo deoxyephedrine or dextromethamphetamine or dexyfed or doe or doxephrin or doxyfed or drinalfa or effroxine or efroxinor efroxine or esophan or estimulex or eufodrin or eufodrinal or gerovit or hiropon or isophan or isophen or kemodrin or madrine or metamfetamine or metamphetamine or methamine or methampex or methamphetamin or methamphetamine or methamphetaminium or methamphin or methedrine or methoxyn or methyl amphetamine or methylamphetamine or methylbenzedrine or methylisamin or methylisomyn or methylpropamine or miller drine or methyl amphetamine or methylamphetamine or n-methylamphetamine or neopharmedrine or normadrine or norodin or norodrin or oxydess or oxydrene or oxyfed or pervitin or phenylisopropylmethylamine hydrochloride or phenylmethylaminopropane hydrochloride or philipon or philopon or premodrin or psykoton or semoxydrine or soxysympamine or syndroxor tonedron) adj2 (abus* or addict* or depend* or habituat* or misus* or mis-us* or problem* or "use disorder" or "use disorders")).tw,kw. (6067)

108 (stimulant? adj2 (abus* or addict* or depend* or habituat* or misus* or mis-us* or problem* or "use disorder" or "use disorders")).tw,kw. (3926)

109 ((psychostimulant? or psycho-stimulant?) adj2 (abus* or addict* or depend* or habituat* or misus* or mis-us* or problem* or "use disorder" or "use disorders")).tw,kw. (2144)

110 ((stimulating or stimulative) adj (agent or agents or drug or drugs) adj2 (abus* or addict* or depend* or habituat* or misus* or mis-us* or problem* or "use disorder" or "use disorders")).tw,kw. (21)

111 (cocaine adj2 (abus* or addict* or depend* or habituat* or misus* or mis-us* or problem* or "use disorder" or "use disorders")).tw,kw. (24141)

112 or/107-111 (34721)

113 84 or 106 or 112 [METHAMPHETAMINE USE DISORDERS/PROBLEMATIC USE] (83547)

114 exp animal/ or exp animal experimentation/ or exp animal model/ or exp animal experiment/ or nonhuman/ or exp vertebrate/ (52797520)

115 exp human/ or exp human experimentation/ or exp human experiment/ (40528425)

116 114 not 115 (12270821)

117 113 not 116 [ANIMAL-ONLY REMOVED] (65251)

118 editorial.pt. (1172654)

119 letter.pt. not (letter.pt. and randomized controlled trial/) (2169504)

120 117 not (118 or 119) [OPINION PIECES REMOVED] (63132)

121 "systematic review"/ (366215)

122 "systematic review (topic)"/ (24707)

123 meta-analysis/ (302450)

124 "meta analysis (topic)"/ (41686)

125 (meta-analy* or metanaly* or metaanaly* or met analy* or integrative research or integrative review* or integrative overview* or research integration or research overview* or collaborative review*).tw,kw. (472800)

126 (systematic review* or systematic overview* or evidence-based review* or evidence-based overview* or (evidence adj3 (review* or overview*)) or meta-review* or meta-overview* or meta-synthes* or rapid review* or "review of reviews" or umbrella review? or technology assessment* or HTA or HTAs).tw,kw. (588369)

127 biomedical technology assessment/ (24564)

128 (cochrane or health technology assessment or evidence report or systematic reviews).jw. (58222)

129 (network adj (MA or MAs)).tw,kw. (28)

130 (NMA or NMAs or MTC or MTCs or MAIC or MAICs).tw,kw. (18191)

131 indirect* compar*.tw,kw. (6634)

132 (indirect treatment* adj1 compar*).tw,kw. (935)

133 (mixed treatment* adj1 compar*).tw,kw. (1525)

134 (multiple treatment* adj1 compar*).tw,kw. (466)

135 (multi-treatment* adj1 compar*).tw,kw. (8)

136 simultaneous* compar*.tw,kw. (2630)

137 mixed comparison?.tw,kw. (144)

138 or/121-137 (1046359)

139 120 and 138 [REVIEWS] (1774)

140 critical pathway/ (15042)

141 clinical protocol/ (125249)

142 consensus/ (81254)

143 consensus development/ (36710)

144 practice guideline/ (438624)

145 health care planning/ (100741)

146 (position statement* or policy statement* or practice parameter* or best practice*).tw,kw. (96976)

147 (standards or guideline or guidelines).ti,kw. (270247)

148 ((practice or treatment* or clinical) adj guideline*).ab. (106289)

149 (CPG or CPGs).ti. (12648)

150 consensus*.ti,kw. (60867)

151 consensus*.ab. /freq=2 (62404)

152 ((critical or clinical or practice) adj2 (path or paths or pathway or pathways or protocol*)).tw,kw. (53241)

153 recommendat*.ti,kw. (98004)

154 (overview? adj2 guideline?).tw,kw. (303)

155 or/140-154 [CPG FILTER] (1228504)

156 120 and 155 [CPGs] (958)

157 139 or 156 [REVIEWS, CPGs] (2617)

158 limit 157 to yr="2015-current" [Limit not valid in DARE; records were retained] (1002)

159 158 use emczd [EMBASE RECORDS] (576)

160 methamphetamine*.ti,ab,kw. (32116)

161 (adipex or ambar or amphedroxyn or cidrin or corvitin or desoxyephedrine hydrochloride or daropervamin or delfetamine stedytab? or deofed or deoxyephedrine or desamine or desfedrin or desoxo or desoxyephedrine or desoxyfed or desoxyn or destim or desyphed or detrex or dextrim or dextro desoxyephedrine or dextro methylamphetamine or dextrolevo deoxyephedrine or dextromethamphetamine or dexyfed or doe or doxephrin or doxyfed or drinalfa or effroxine or efroxinor efroxine or esophan or estimulex or eufodrin or eufodrinal or gerovit or hiropon or isophan or isophen or kemodrin or madrine or metamfetamine or metamphetamine or methamine or methampex or methamphetamin or methamphetaminium or methamphin or methedrine or methoxyn or methyl amphetamine or methylamphetamine or methylbenzedrine or methylisamin or methylisomyn or methylpropamine or miller drine or methyl amphetamine or methylamphetamine or n-methylamphetamine or neopharmedrine or normadrine or norodin or norodrin or oxydess or oxydrene or oxyfed or pervitin or phenylisopropylmethylamine hydrochloride or phenylmethylaminopropane hydrochloride or philipon or philopon or premodrin or psykoton or semoxydrine or soxysympamine or syndroxor tonedron).ti,ab,kw. (10670)

162 (crystal meth* or crank).ti,ab,kw. (3459)

163 uppers.ti,ab,kw. (169)

164 (central nervous system stimulant? or CNS stimulant?).ti,ab,kw. (2669)

165 (psychostimulant? or psycho-stimulant?).ti,ab,kw. (18783)

166 ((stimulant? or stimulating or stimulative) adj (agent or agents or drug or drugs)).ti,ab,kw. (14830)

167 cocaine.ti,ab,kw. (107507)

168 or/160-167 [METHAMPHETAMINE/CNS STIMULANTS/COCAINE] (173544)

169 ((drug? or drug-related) adj2 (abus* or addict* or depend* or habituat* or misus* or mis-us* or problem* or "use disorder" or "use disorders")).ti,ab,kw. (179837)

170 drug-related disorder?.ti,ab,kw. (196)

171 ((substance* or substance-related) adj2 (abus* or addict* or depend* or habituat* or misus* or mis-us* or problem* or "use disorder" or "use disorders")).ti,ab,kw. (161008)

172 substance-related disorder?.ti,ab,kw. (3588)

173 (MUD or MUDs).ti,ab,kw. (14904)

174 (PMU or PMUs).ti,ab,kw. (559)

175 or/169-174 [SUBSTANCE DISORDERS] (334873)

176 168 and 175 [METHAMPHETAMINE DISORDERS/PROBLEMATIC USE] (36257)

177 ((methamphetamine* or adipex or ambar or amphedroxyn or cidrin or corvitin or desoxyephedrine hydrochloride or daropervamin or delfetamine stedytab? or deofed or deoxyephedrine or desamine or desfedrin or desoxo or desoxyephedrine or desoxyfed or desoxyn or destim or desyphed or detrex or dextrim or dextro desoxyephedrine or dextro methylamphetamine or dextrolevo deoxyephedrine or dextromethamphetamine or dexyfed or doe or doxephrin or doxyfed or drinalfa or effroxine or efroxinor efroxine or esophan or estimulex or eufodrin or eufodrinal or gerovit or hiropon or isophan or isophen or kemodrin or madrine or metamfetamine or metamphetamine or methamine or methampex or methamphetamin or methamphetamine or methamphetaminium or methamphin or methedrine or methoxyn or methyl amphetamine or methylamphetamine or methylbenzedrine or methylisamin or methylisomyn or methylpropamine or miller drine or methyl amphetamine or methylamphetamine or n-methylamphetamine or neopharmedrine or normadrine or norodin or norodrin or oxydess or oxydrene or oxyfed or pervitin or phenylisopropylmethylamine hydrochloride or phenylmethylaminopropane hydrochloride or philipon or philopon or premodrin or psykoton or semoxydrine or soxysympamine or syndroxor tonedron) adj2 (abus* or addict* or depend* or habituat* or misus* or mis-us* or problem* or "use disorder" or "use disorders")).ti,ab,kw. (6008)

178 (stimulant? adj2 (abus* or addict* or depend* or habituat* or misus* or mis-us* or problem* or "use disorder" or "use disorders")).ti,ab,kw. (3842)

179 ((psychostimulant? or psycho-stimulant?) adj2 (abus* or addict* or depend* or habituat* or misus* or mis-us* or problem* or "use disorder" or "use disorders")).ti,ab,kw. (2121)

180 ((stimulating or stimulative) adj (agent or agents or drug or drugs) adj2 (abus* or addict* or depend* or habituat* or misus* or mis-us* or problem* or "use disorder" or "use disorders")).ti,ab,kw. (21)

181 (cocaine adj2 (abus* or addict* or depend* or habituat* or misus* or mis-us* or problem* or "use disorder" or "use disorders")).ti,ab,kw. (23769)

182 or/177-181 (34246)

183 176 or 182 [METHAMPHETAMINE USE DISORDERS/PROBLEMATIC USE] (59098)

184 limit 183 to yr="2015-current" [Limit not valid in DARE; records were retained] (15198)

185 184 use coch,dare,clhta [COCHRANE RECORDS] (46)

186 Methamphetamine/ (33144)

187 methamphetamine*.tw,id. (31865)

188 (adipex or ambar or amphedroxyn or cidrin or corvitin or desoxyephedrine hydrochloride or daropervamin or delfetamine stedytab? or deofed or deoxyephedrine or desamine or desfedrin or desoxo or desoxyephedrine or desoxyfed or desoxyn or destim or desyphed or detrex or dextrim or dextro desoxyephedrine or dextro methylamphetamine or dextrolevo deoxyephedrine or dextromethamphetamine or dexyfed or doe or doxephrin or doxyfed or drinalfa or effroxine or efroxinor efroxine or esophan or estimulex or eufodrin or eufodrinal or gerovit or hiropon or isophan or isophen or kemodrin or madrine or metamfetamine or metamphetamine or methamine or methampex or methamphetamin or methamphetaminium or methamphin or methedrine or methoxyn or methyl amphetamine or methylamphetamine or methylbenzedrine or methylisamin or methylisomyn or methylpropamine or miller drine or methyl amphetamine or methylamphetamine or n-methylamphetamine or neopharmedrine or normadrine or norodin or norodrin or oxydess or oxydrene or oxyfed or pervitin or phenylisopropylmethylamine hydrochloride or phenylmethylaminopropane hydrochloride or philipon or philopon or premodrin or psykoton or semoxydrine or soxysympamine or syndroxor tonedron).tw,id. (10894)

189 (crystal meth* or crank).tw,id. (3428)

190 uppers.tw,id. (172)

191 CNS Stimulating Drugs/ (3015)

192 (central nervous system stimulant? or CNS stimulant?).tw,id. (2540)

193 (psychostimulant? or psycho-stimulant?).tw,id. (18251)

194 ((stimulant? or stimulating or stimulative) adj (agent or agents or drug or drugs)).tw,id. (14835)

195 exp Cocaine/ (96458)

196 cocaine.tw,id. (106839)

197 or/186-196 [METHAMPHETAMINE/CNS STIMULANTS/COCAINE] (202294)

198 Drug Abuse/ (194040)

199 Drug Addiction/ (163169)

200 "Substance Use Disorder"/ (146222)

201 "Substance Use Treatment"/ (20904)

202 ((drug? or drug-related) adj2 (abus* or addict* or depend* or habituat* or misus* or mis-us* or problem* or "use disorder" or "use disorders")).tw,id. (182906)

203 drug-related disorder?.tw,id. (192)

204 ((substance* or substance-related) adj2 (abus* or addict* or depend* or habituat* or misus* or mis-us* or problem* or "use disorder" or "use disorders")).tw,id. (161253)

205 substance-related disorder?.tw,id. (2626)

206 (MUD or MUDs).tw,id. (14828)

207 (PMU or PMUs).tw,id. (557)

208 or/198-207 [SUBSTANCE DISORDERS] (475308)

209 197 and 208 [METHAMPHETAMINE DISORDERS/PROBLEMATIC USE] (61582)

210 ((methamphetamine* or adipex or ambar or amphedroxyn or cidrin or corvitin or desoxyephedrine hydrochloride or daropervamin or delfetamine stedytab? or deofed or deoxyephedrine or desamine or desfedrin or desoxo or desoxyephedrine or desoxyfed or desoxyn or destim or desyphed or detrex or dextrim or dextro desoxyephedrine or dextro methylamphetamine or dextrolevo deoxyephedrine or dextromethamphetamine or dexyfed or doe or doxephrin or doxyfed or drinalfa or effroxine or efroxinor efroxine or esophan or estimulex or eufodrin or eufodrinal or gerovit or hiropon or isophan or isophen or kemodrin or madrine or metamfetamine or metamphetamine or methamine or methampex or methamphetamin or methamphetamine or methamphetaminium or methamphin or methedrine or methoxyn or methyl amphetamine or methylamphetamine or methylbenzedrine or methylisamin or methylisomyn or methylpropamine or miller drine or methyl amphetamine or methylamphetamine or n-methylamphetamine or neopharmedrine or normadrine or norodin or norodrin or oxydess or oxydrene or oxyfed or pervitin or phenylisopropylmethylamine hydrochloride or phenylmethylaminopropane hydrochloride or philipon or philopon or premodrin or psykoton or semoxydrine or soxysympamine or syndroxor tonedron) adj2 (abus* or addict* or depend* or habituat* or misus* or mis-us* or problem* or "use disorder" or "use disorders")).tw,id. (5983)

211 (stimulant? adj2 (abus* or addict* or depend* or habituat* or misus* or mis-us* or problem* or "use disorder" or "use disorders")).tw,id. (3843)

212 ((psychostimulant? or psycho-stimulant?) adj2 (abus* or addict* or depend* or habituat* or misus* or mis-us* or problem* or "use disorder" or "use disorders")).tw,id. (2061)

213 ((stimulating or stimulative) adj (agent or agents or drug or drugs) adj2 (abus* or addict* or depend* or habituat* or misus* or mis-us* or problem* or "use disorder" or "use disorders")).tw,id. (21)

214 (cocaine adj2 (abus* or addict* or depend* or habituat* or misus* or mis-us* or problem* or "use disorder" or "use disorders")).tw,id. (23768)

215 or/210-214 (34205)

216 209 or 215 [METHAMPHETAMINE USE DISORDERS/PROBLEMATIC USE] (78560)

217 Systematic Review/ (366215)

218 Meta Analysis/ (302450)

219 (meta-analy* or metanaly* or metaanaly* or met analy* or integrative research or integrative review* or integrative overview* or research integration or research overview* or collaborative review*).tw,id. (468209)

220 (systematic review* or systematic overview* or evidence-based review* or evidence-based overview* or (evidence adj3 (review* or overview*)) or meta-review* or meta-overview* or meta-synthes* or rapid review* or "review of reviews" or umbrella review? or technology assessment* or HTA or HTAs).tw,id. (582480)

221 (network adj (MA or MAs)).tw,id. (28)

222 (NMA or NMAs or MTC or MTCs or MAIC or MAICs).tw,id. (18089)

223 indirect* compar*.tw,id. (6530)

224 (indirect treatment* adj1 compar*).tw,id. (907)

225 (mixed treatment* adj1 compar*).tw,id. (1439)

226 (multiple treatment* adj1 compar*).tw,id. (446)

227 (multi-treatment* adj1 compar*).tw,id. (8)

228 simultaneous* compar*.tw,id. (2630)

229 mixed comparison?.tw,id. (143)

230 or/217-229 (979936)

231 216 and 230 [REVIEWS] (1586)

232 Treatment Guidelines/ (6804)

233 (position statement* or policy statement* or practice parameter* or best practice*).tw,id. (96129)

234 (standards or guideline or guidelines).ti,id. (251476)

235 ((practice or treatment* or clinical) adj guideline*).ab. (106289)

236 (CPG or CPGs).ti. (12648)

237 consensus*.ti,id. (57582)

238 consensus*.ab. /freq=2 (62404)

239 ((critical or clinical or practice) adj2 (path or paths or pathway or pathways or protocol*)).tw,id. (52226)

240 recommendat*.ti,id. (95687)

241 (overview? adj2 guideline?).tw,id. (297)

242 or/232-241 [CPG FILTER] (652502)

243 216 and 242 [CPGs] (439)

244 231 or 243 [REVIEWS, CPGs] (1975)

245 limit 244 to yr="2015-current" [Limit not valid in DARE; records were retained] (788)

246 245 use medall,emczd,coch,dare,clhta (650)

247 245 not 246 [PSYCINFO RECORDS] (138)

248 79 or 159 or 185 or 247 [ALL DATABASES] (1004)

249 conference abstract.pt. (3747631)

250 248 not 249 [CONFERENCE ABSTRACTS REMOVED] (878)

251 remove duplicates from 250 (597) [TOTAL UNIQUE RECORDS]

252 251 use medall [MEDLINE UNIQUE RECORDS] (241)

253 251 use emczd [EMBASE UNIQUE RECORDS] (275)

254 251 use coch [COCHRANE DSR UNIQUE RECORDS] (9)

255 251 use dare [DARE UNIQUE RECORDS] (37)

256 251 use clhta [HTA UNIQUE RECORDS] (0)

257 251 not (252 or 253 or 254 or 255 or 256) [PSYCINFO RECORDS] (35)

*Primary Studies*

Database: Embase Classic+Embase <1947 to 2020 April 13>, Ovid MEDLINE(R) ALL <1946 to April 13, 2020>, APA PsycInfo <1806 to April Week 1 2020>, EBM Reviews - Cochrane Central Register of Controlled Trials <February 2020>

Search Strategy:

--------------------------------------------------------------------------------

1 Amphetamine-Related Disorders/ (3657)

2 Cocaine-Related Disorders/ (15243)

3 1 or 2 [AMPHETAMINE/COCAINE-RELATED DISORDERS] (18423)

4 Methamphetamine/ (33690)

5 methamphetamine*.tw,kf,rn. (41494)

6 (adipex or ambar or amphedroxyn or cidrin or corvitin or desoxyephedrine hydrochloride or daropervamin or delfetamine stedytab? or deofed or deoxyephedrine or desamine or desfedrin or desoxo or desoxyephedrine or desoxyfed or desoxyn or destim or desyphed or detrex or dextrim or dextro desoxyephedrine or dextro methylamphetamine or dextrolevo deoxyephedrine or dextromethamphetamine or dexyfed or doe or doxephrin or doxyfed or drinalfa or effroxine or efroxinor efroxine or esophan or estimulex or eufodrin or eufodrinal or gerovit or hiropon or isophan or isophen or kemodrin or madrine or metamfetamine or metamphetamine or methamine or methampex or methamphetamin or methamphetaminium or methamphin or methedrine or methoxyn or methyl amphetamine or methylamphetamine or methylbenzedrine or methylisamin or methylisomyn or methylpropamine or miller drine or methyl amphetamine or methylamphetamine or n-methylamphetamine or neopharmedrine or normadrine or norodin or norodrin or oxydess or oxydrene or oxyfed or pervitin or phenylisopropylmethylamine hydrochloride or phenylmethylaminopropane hydrochloride or philipon or philopon or premodrin or psykoton or semoxydrine or soxysympamine or syndroxor tonedron).tw,kf. (11252)

7 (crystal meth* or crank).tw,kf. (3588)

8 uppers.tw,kf. (174)

9 Central Nervous System Stimulants/ (40392)

10 (central nervous system stimulant? or CNS stimulant?).tw,kf. (2568)

11 (psychostimulant? or psycho-stimulant?).tw,kf. (18902)

12 ((stimulant? or stimulating or stimulative) adj (agent or agents or drug or drugs)).tw,kf. (15767)

13 exp Cocaine/ (97404)

14 cocaine.tw,kf. (110360)

15 or/4-14 [METHAMPHETAMINE/CNS STIMULANTS/COCAINE] (237257)

16 Substance-Related Disorders/ (124626)

17 ((drug? or drug-related) adj2 (abus* or addict* or depend* or habituat* or misus* or mis-us* or problem* or "use disorder" or "use disorders")).tw,kf. (190599)

18 drug-related disorder?.tw,kf. (201)

19 ((substance* or substance-related) adj2 (abus* or addict* or depend* or habituat* or misus* or mis-us* or problem* or "use disorder" or "use disorders")).tw,kf. (168131)

20 substance-related disorder?.tw,kf. (2829)

21 (MUD or MUDs).tw,kf. (15240)

22 (PMU or PMUs).tw,kf. (578)

23 or/16-22 [SUBSTANCE DISORDERS] (423482)

24 15 and 23 [METHAMPHETAMINE DISORDERS/PROBLEMATIC USE] (52931)

25 ((methamphetamine* or adipex or ambar or amphedroxyn or cidrin or corvitin or desoxyephedrine hydrochloride or daropervamin or delfetamine stedytab? or deofed or deoxyephedrine or desamine or desfedrin or desoxo or desoxyephedrine or desoxyfed or desoxyn or destim or desyphed or detrex or dextrim or dextro desoxyephedrine or dextro methylamphetamine or dextrolevo deoxyephedrine or dextromethamphetamine or dexyfed or doe or doxephrin or doxyfed or drinalfa or effroxine or efroxinor efroxine or esophan or estimulex or eufodrin or eufodrinal or gerovit or hiropon or isophan or isophen or kemodrin or madrine or metamfetamine or metamphetamine or methamine or methampex or methamphetamin or methamphetamine or methamphetaminium or methamphin or methedrine or methoxyn or methyl amphetamine or methylamphetamine or methylbenzedrine or methylisamin or methylisomyn or methylpropamine or miller drine or methyl amphetamine or methylamphetamine or n-methylamphetamine or neopharmedrine or normadrine or norodin or norodrin or oxydess or oxydrene or oxyfed or pervitin or phenylisopropylmethylamine hydrochloride or phenylmethylaminopropane hydrochloride or philipon or philopon or premodrin or psykoton or semoxydrine or soxysympamine or syndroxor tonedron) adj2 (abus* or addict* or depend* or habituat* or misus* or mis-us* or problem* or "use disorder" or "use disorders")).tw,kf. (6482)

26 (stimulant? adj2 (abus* or addict* or depend* or habituat* or misus* or mis-us* or misus* or mis-us* or problem* or "use disorder" or "use disorders")).tw,kf. (4170)

27 ((psychostimulant? or psycho-stimulant?) adj2 (abus* or addict* or depend* or habituat* or misus* or mis-us* or problem* or "use disorder" or "use disorders")).tw,kf. (2106)

28 ((stimulating or stimulative) adj (agent or agents or drug or drugs) adj2 (abus* or addict* or depend* or habituat* or misus* or mis-us* or problem* or "use disorder" or "use disorders")).tw,kf. (23)

29 (cocaine adj2 (abus* or addict* or depend* or habituat* or misus* or mis-us* or problem* or "use disorder" or "use disorders")).tw,kf. (25642)

30 or/25-29 (36789)

31 3 or 24 or 30 [METHAMPHETAMINE USE DISORDERS/PROBLEMATIC USE] (83334)

32 exp Animals/ not Humans/ (18167289)

33 31 not 32 [ANIMAL-ONLY REMOVED] (57688)

34 (comment or editorial or news or newspaper article).pt. (2054759)

35 (letter not (letter and randomized controlled trial)).pt. (2176727)

36 33 not (34 or 35) [OPINION PIECES REMOVED] (55597)

37 (controlled clinical trial or randomized controlled trial or pragmatic clinical trial or equivalence trial).pt. (1170623)

38 clinical trials as topic/ (301596)

39 exp "Controlled Clinical Trials as Topic"/ (332397)

40 (randomi#ed or randomi#ation? or randomly or RCT or placebo*).tw,kf. (3516751)

41 ((singl* or doubl* or trebl* or tripl*) adj (mask* or blind* or dumm*)).tw,kf. (708209)

42 trial.ti. (858507)

43 or/37-42 (4435599)

44 36 and 43 [RCTs] (8461)

45 controlled clinical trial.pt. (185004)

46 Controlled Clinical Trial/ or Controlled Clinical Trials as Topic/ (572546)

47 (control* adj2 trial).tw,kf. (629623)

48 Non-Randomized Controlled Trials as Topic/ (11348)

49 (nonrandom* or non-random* or quasi-random* or quasi-experiment*).tw,kf. (156416)

50 (nRCT or non-RCT).tw,kf. (884)

51 Controlled Before-After Studies/ (217936)

52 (control* adj3 ("before and after" or "before after")).tw,kf. (724790)

53 Interrupted Time Series Analysis/ (211015)

54 time series.tw,kf. (73917)

55 (pre- adj3 post-).tw,kf. (284587)

56 (pretest adj3 posttest).tw,kf. (22043)

57 Historically Controlled Study/ (228424)

58 (control* adj2 study).tw,kf. (526535)

59 Control Groups/ (125854)

60 (control* adj2 group?).tw,kf. (1518350)

61 trial.ti. (858507)

62 or/45-61 (4178766)

63 36 and 62 [non-RCTs] (6300)

64 exp Cohort Studies/ (2692016)

65 cohort?.tw,kf. (1682955)

66 Retrospective Studies/ (1450969)

67 (longitudinal or prospective or retrospective).tw,kf. (3569398)

68 ((followup or follow-up) adj (study or studies)).tw,kf. (142532)

69 Observational study.pt. (78683)

70 (observation$2 adj (study or studies)).tw,kf. (292756)

71 ((population or population-based) adj (study or studies or analys#s)).tw,kf. (46133)

72 Comparative Study.pt. (2024654)

73 ((comparative or comparison) adj (study or studies)).tw,kf. (297921)

74 or/64-73 (8029850)

75 36 and 74 [OBSERVATIONAL STUDIES] (8697)

76 44 or 63 or 75 [ALL PRIMARY STUDY DESIGNS] (17207)

77 76 use medall [MEDLINE RECORDS] (7657)

78 methamphetamine dependence/ (1477)

79 methamphetamine-induced psychosis/ (77)

80 cocaine dependence/ (21754)

81 cocaine-induced psychosis/ (14)

82 or/78-81 [AMPHETAMINE/COCAINE-RELATED DISORDERS] (22954)

83 Methamphetamine/ (33690)

84 methamphetamine*.tw,kw,rn. (41655)

85 (adipex or ambar or amphedroxyn or cidrin or corvitin or desoxyephedrine hydrochloride or daropervamin or delfetamine stedytab? or deofed or deoxyephedrine or desamine or desfedrin or desoxo or desoxyephedrine or desoxyfed or desoxyn or destim or desyphed or detrex or dextrim or dextro desoxyephedrine or dextro methylamphetamine or dextrolevo deoxyephedrine or dextromethamphetamine or dexyfed or doe or doxephrin or doxyfed or drinalfa or effroxine or efroxinor efroxine or esophan or estimulex or eufodrin or eufodrinal or gerovit or hiropon or isophan or isophen or kemodrin or madrine or metamfetamine or metamphetamine or methamine or methampex or methamphetamin or methamphetaminium or methamphin or methedrine or methoxyn or methyl amphetamine or methylamphetamine or methylbenzedrine or methylisamin or methylisomyn or methylpropamine or miller drine or methyl amphetamine or methylamphetamine or n-methylamphetamine or neopharmedrine or normadrine or norodin or norodrin or oxydess or oxydrene or oxyfed or pervitin or phenylisopropylmethylamine hydrochloride or phenylmethylaminopropane hydrochloride or philipon or philopon or premodrin or psykoton or semoxydrine or soxysympamine or syndroxor tonedron).tw,kw. (11287)

86 (crystal meth* or crank).tw,kw. (3635)

87 uppers.tw,kw. (174)

88 central stimulant agent/ (19256)

89 psychostimulant agent/ (5795)

90 (central nervous system stimulant? or CNS stimulant?).tw,kw. (2711)

91 (psychostimulant? or psycho-stimulant?).tw,kw. (19551)

92 ((stimulant? or stimulating or stimulative) adj (agent or agents or drug or drugs)).tw,kw. (16667)

93 cocaine/ (95669)

94 cocaine.tw,kw. (111259)

95 or/83-94 [METHAMPHETAMINE/CNS STIMULANTS/COCAINE] (226027)

96 drug dependence/ (155537)

97 ((drug? or drug-related) adj2 (abus* or addict* or depend* or habituat* or misus* or mis-us* or problem* or "use disorder" or "use disorders")).tw,kw. (194634)

98 drug-related disorder?.tw,kw. (205)

99 ((substance* or substance-related) adj2 (abus* or addict* or depend* or habituat* or misus* or mis-us* or problem* or "use disorder" or "use disorders")).tw,kw. (170713)

100 substance-related disorder?.tw,kw. (3870)

101 (MUD or MUDs).tw,kw. (15198)

102 (PMU or PMUs).tw,kw. (580)

103 or/96-102 [SUBSTANCE DISORDERS] (445002)

104 95 and 103 [METHAMPHETAMINE DISORDERS/PROBLEMATIC USE] (56601)

105 ((methamphetamine* or adipex or ambar or amphedroxyn or cidrin or corvitin or desoxyephedrine hydrochloride or daropervamin or delfetamine stedytab? or deofed or deoxyephedrine or desamine or desfedrin or desoxo or desoxyephedrine or desoxyfed or desoxyn or destim or desyphed or detrex or dextrim or dextro desoxyephedrine or dextro methylamphetamine or dextrolevo deoxyephedrine or dextromethamphetamine or dexyfed or doe or doxephrin or doxyfed or drinalfa or effroxine or efroxinor efroxine or esophan or estimulex or eufodrin or eufodrinal or gerovit or hiropon or isophan or isophen or kemodrin or madrine or metamfetamine or metamphetamine or methamine or methampex or methamphetamin or methamphetamine or methamphetaminium or methamphin or methedrine or methoxyn or methyl amphetamine or methylamphetamine or methylbenzedrine or methylisamin or methylisomyn or methylpropamine or miller drine or methyl amphetamine or methylamphetamine or n-methylamphetamine or neopharmedrine or normadrine or norodin or norodrin or oxydess or oxydrene or oxyfed or pervitin or phenylisopropylmethylamine hydrochloride or phenylmethylaminopropane hydrochloride or philipon or philopon or premodrin or psykoton or semoxydrine or soxysympamine or syndroxor tonedron) adj2 (abus* or addict* or depend* or habituat* or misus* or mis-us* or problem* or "use disorder" or "use disorders")).tw,kw. (6571)

106 (stimulant? adj2 (abus* or addict* or depend* or habituat* or misus* or mis-us* or problem* or "use disorder" or "use disorders")).tw,kw. (4229)

107 ((psychostimulant? or psycho-stimulant?) adj2 (abus* or addict* or depend* or habituat* or misus* or mis-us* or problem* or "use disorder" or "use disorders")).tw,kw. (2184)

108 ((stimulating or stimulative) adj (agent or agents or drug or drugs) adj2 (abus* or addict* or depend* or habituat* or misus* or mis-us* or problem* or "use disorder" or "use disorders")).tw,kw. (23)

109 (cocaine adj2 (abus* or addict* or depend* or habituat* or misus* or mis-us* or problem* or "use disorder" or "use disorders")).tw,kw. (26070)

110 or/105-109 (37338)

111 82 or 104 or 110 [METHAMPHETAMINE USE DISORDERS/PROBLEMATIC USE] (86888)

112 exp animal/ or exp animal experimentation/ or exp animal model/ or exp animal experiment/ or nonhuman/ or exp vertebrate/ (53371983)

113 exp human/ or exp human experimentation/ or exp human experiment/ (41102900)

114 112 not 113 (12270809)

115 111 not 114 [ANIMAL-ONLY REMOVED] (68592)

116 editorial.pt. (1173109)

117 letter.pt. not (letter.pt. and randomized controlled trial/) (2176595)

118 115 not (116 or 117) [OPINION PIECES REMOVED] (66460)

119 exp randomized controlled trial/ or controlled clinical trial/ (1382013)

120 clinical trial/ (1526976)

121 exp "controlled clinical trial (topic)"/ (184350)

122 (randomi#ed or randomi#ation? or randomly or RCT or placebo*).tw,kw. (3571969)

123 ((singl* or doubl* or trebl* or tripl*) adj (mask* or blind* or dumm*)).tw,kw. (733029)

124 trial.ti. (858507)

125 or/119-124 [RCT FILTER] (5017088)

126 118 and 125 [RCTs] (10650)

127 controlled clinical trial/ (558002)

128 "controlled clinical trial (topic)"/ (10671)

129 (control* adj2 trial).tw,kw. (936847)

130 (nonrandom* or non-random* or quasi-random* or quasi-experiment*).tw,kw. (156766)

131 (nRCT or nonRCT or non-RCT).tw,kw. (897)

132 (control* adj3 ("before and after" or "before after")).tw,kw. (724795)

133 time series analysis/ (25672)

134 time series.tw,kw. (74841)

135 pretest posttest control group design/ (455)

136 (pre- adj3 post-).tw,kw. (284629)

137 (pretest adj3 posttest).tw,kw. (22877)

138 controlled study/ (7353919)

139 (control* adj2 study).tw,kw. (874757)

140 control group/ (125758)

141 (control* adj2 group?).tw,kw. (1518576)

142 trial.ti. (858507)

143 or/127-142 [nRCT FILTER] (10323422)

144 118 and 143 [nRCTs] (13281)

145 cohort analysis/ (827222)

146 cohort?.tw,kw. (1687867)

147 retrospective study/ (1717189)

148 longitudinal study/ (271742)

149 prospective study/ (1130029)

150 (longitudinal or prospective or retrospective).tw,kw. (3585289)

151 follow up/ (1566327)

152 ((followup or follow-up) adj (study or studies)).tw,kw. (144345)

153 observational study/ (271304)

154 (observation$2 adj (study or studies)).tw,kw. (295067)

155 population research/ (106897)

156 ((population or population-based) adj (study or studies or analys#s)).tw,kw. (54217)

157 exp comparative study/ (3288847)

158 ((comparative or comparison) adj (study or studies)).tw,kw. (312525)

159 or/145-158 [OBSERVATIONAL STUDY FILTER] (9921628)

160 118 and 159 [OBSERVATIONAL STUDIES] (11661)

161 126 or 144 or 160 (25744)

162 126 or 144 or 160 [ALL STUDY DESIGNS] (25744)

163 conference abstract.pt. (3764397)

164 162 not 163 [CONFERENCE ABSTRACTS REMOVED] (23927)

165 164 use emczd [EMBASE RECORDS] (11808)

166 Amphetamine-Related Disorders/ (3657)

167 Cocaine-Related Disorders/ (15243)

168 166 or 167 [AMPHETAMINE/COCAINE-RELATED DISORDERS] (18423)

169 Methamphetamine/ (33690)

170 methamphetamine*.ti,ab,kw. (33034)

171 (adipex or ambar or amphedroxyn or cidrin or corvitin or desoxyephedrine hydrochloride or daropervamin or delfetamine stedytab? or deofed or deoxyephedrine or desamine or desfedrin or desoxo or desoxyephedrine or desoxyfed or desoxyn or destim or desyphed or detrex or dextrim or dextro desoxyephedrine or dextro methylamphetamine or dextrolevo deoxyephedrine or dextromethamphetamine or dexyfed or doe or doxephrin or doxyfed or drinalfa or effroxine or efroxinor efroxine or esophan or estimulex or eufodrin or eufodrinal or gerovit or hiropon or isophan or isophen or kemodrin or madrine or metamfetamine or metamphetamine or methamine or methampex or methamphetamin or methamphetaminium or methamphin or methedrine or methoxyn or methyl amphetamine or methylamphetamine or methylbenzedrine or methylisamin or methylisomyn or methylpropamine or miller drine or methyl amphetamine or methylamphetamine or n-methylamphetamine or neopharmedrine or normadrine or norodin or norodrin or oxydess or oxydrene or oxyfed or pervitin or phenylisopropylmethylamine hydrochloride or phenylmethylaminopropane hydrochloride or philipon or philopon or premodrin or psykoton or semoxydrine or soxysympamine or syndroxor tonedron).ti,ab,kw. (10790)

172 (crystal meth* or crank).ti,ab,kw. (3619)

173 uppers.ti,ab,kw. (172)

174 Central Nervous System Stimulants/ (40392)

175 (central nervous system stimulant? or CNS stimulant?).ti,ab,kw. (2663)

176 (psychostimulant? or psycho-stimulant?).ti,ab,kw. (19307)

177 ((stimulant? or stimulating or stimulative) adj (agent or agents or drug or drugs)).ti,ab,kw. (16572)

178 exp Cocaine/ (97404)

179 cocaine.ti,ab,kw. (110750)

180 or/169-179 [METHAMPHETAMINE/CNS STIMULANTS/COCAINE] (238310)

181 Substance-Related Disorders/ (124626)

182 ((drug? or drug-related) adj2 (abus* or addict* or depend* or habituat* or misus* or mis-us* or problem* or "use disorder" or "use disorders")).ti,ab,kw. (187676)

183 drug-related disorder?.ti,ab,kw. (200)

184 ((substance* or substance-related) adj2 (abus* or addict* or depend* or habituat* or misus* or mis-us* or problem* or "use disorder" or "use disorders")).ti,ab,kw. (166930)

185 substance-related disorder?.ti,ab,kw. (3466)

186 (MUD or MUDs).ti,ab,kw. (15181)

187 (PMU or PMUs).ti,ab,kw. (579)

188 or/181-187 [SUBSTANCE DISORDERS] (421340)

189 180 and 188 [METHAMPHETAMINE DISORDERS/PROBLEMATIC USE] (53740)

190 ((methamphetamine* or adipex or ambar or amphedroxyn or cidrin or corvitin or desoxyephedrine hydrochloride or daropervamin or delfetamine stedytab? or deofed or deoxyephedrine or desamine or desfedrin or desoxo or desoxyephedrine or desoxyfed or desoxyn or destim or desyphed or detrex or dextrim or dextro desoxyephedrine or dextro methylamphetamine or dextrolevo deoxyephedrine or dextromethamphetamine or dexyfed or doe or doxephrin or doxyfed or drinalfa or effroxine or efroxinor efroxine or esophan or estimulex or eufodrin or eufodrinal or gerovit or hiropon or isophan or isophen or kemodrin or madrine or metamfetamine or metamphetamine or methamine or methampex or methamphetamin or methamphetamine or methamphetaminium or methamphin or methedrine or methoxyn or methyl amphetamine or methylamphetamine or methylbenzedrine or methylisamin or methylisomyn or methylpropamine or miller drine or methyl amphetamine or methylamphetamine or n-methylamphetamine or neopharmedrine or normadrine or norodin or norodrin or oxydess or oxydrene or oxyfed or pervitin or phenylisopropylmethylamine hydrochloride or phenylmethylaminopropane hydrochloride or philipon or philopon or premodrin or psykoton or semoxydrine or soxysympamine or syndroxor tonedron) adj2 (abus* or addict* or depend* or habituat* or misus* or mis-us* or problem* or "use disorder" or "use disorders")).ti,ab,kw. (6518)

191 (stimulant? adj2 (abus* or addict* or depend* or habituat* or misus* or mis-us* or misus* or mis-us* or problem* or "use disorder" or "use disorders")).ti,ab,kw. (4160)

192 ((psychostimulant? or psycho-stimulant?) adj2 (abus* or addict* or depend* or habituat* or misus* or mis-us* or problem* or "use disorder" or "use disorders")).ti,ab,kw. (2169)

193 ((stimulating or stimulative) adj (agent or agents or drug or drugs) adj2 (abus* or addict* or depend* or habituat* or misus* or mis-us* or problem* or "use disorder" or "use disorders")).ti,ab,kw. (23)

194 (cocaine adj2 (abus* or addict* or depend* or habituat* or misus* or mis-us* or problem* or "use disorder" or "use disorders")).ti,ab,kw. (25729)

195 or/190-194 (36907)

196 168 or 189 or 195 [METHAMPHETAMINE USE DISORDERS/PROBLEMATIC USE] (83891)

197 conference abstract.pt. (3764397)

198 196 not 197 [CONFERENCE ABSTRACTS REMOVED] (78821)

199 198 use cctr [CENTRAL RECORDS] (3686)

200 Methamphetamine/ (33690)

201 methamphetamine*.tw,id. (32720)

202 (adipex or ambar or amphedroxyn or cidrin or corvitin or desoxyephedrine hydrochloride or daropervamin or delfetamine stedytab? or deofed or deoxyephedrine or desamine or desfedrin or desoxo or desoxyephedrine or desoxyfed or desoxyn or destim or desyphed or detrex or dextrim or dextro desoxyephedrine or dextro methylamphetamine or dextrolevo deoxyephedrine or dextromethamphetamine or dexyfed or doe or doxephrin or doxyfed or drinalfa or effroxine or efroxinor efroxine or esophan or estimulex or eufodrin or eufodrinal or gerovit or hiropon or isophan or isophen or kemodrin or madrine or metamfetamine or metamphetamine or methamine or methampex or methamphetamin or methamphetaminium or methamphin or methedrine or methoxyn or methyl amphetamine or methylamphetamine or methylbenzedrine or methylisamin or methylisomyn or methylpropamine or miller drine or methyl amphetamine or methylamphetamine or n-methylamphetamine or neopharmedrine or normadrine or norodin or norodrin or oxydess or oxydrene or oxyfed or pervitin or phenylisopropylmethylamine hydrochloride or phenylmethylaminopropane hydrochloride or philipon or philopon or premodrin or psykoton or semoxydrine or soxysympamine or syndroxor tonedron).tw,id. (10990)

203 (crystal meth* or crank).tw,id. (3572)

204 uppers.tw,id. (174)

205 CNS Stimulating Drugs/ (3015)

206 (central nervous system stimulant? or CNS stimulant?).tw,id. (2509)

207 (psychostimulant? or psycho-stimulant?).tw,id. (18708)

208 ((stimulant? or stimulating or stimulative) adj (agent or agents or drug or drugs)).tw,id. (15705)

209 exp Cocaine/ (97404)

210 cocaine.tw,id. (109868)

211 or/200-210 [METHAMPHETAMINE/CNS STIMULANTS/COCAINE] (207818)

212 Drug Abuse/ (197793)

213 Drug Addiction/ (166922)

214 "Substance Use Disorder"/ (149975)

215 "Substance Use Treatment"/ (20904)

216 ((drug? or drug-related) adj2 (abus* or addict* or depend* or habituat* or misus* or mis-us* or problem* or "use disorder" or "use disorders")).tw,id. (188452)

217 drug-related disorder?.tw,id. (196)

218 ((substance* or substance-related) adj2 (abus* or addict* or depend* or habituat* or misus* or mis-us* or problem* or "use disorder" or "use disorders")).tw,id. (166321)

219 substance-related disorder?.tw,id. (2377)

220 (MUD or MUDs).tw,id. (15085)

221 (PMU or PMUs).tw,id. (577)

222 or/212-221 [SUBSTANCE DISORDERS] (487302)

223 211 and 222 [METHAMPHETAMINE DISORDERS/PROBLEMATIC USE] (62813)

224 ((methamphetamine* or adipex or ambar or amphedroxyn or cidrin or corvitin or desoxyephedrine hydrochloride or daropervamin or delfetamine stedytab? or deofed or deoxyephedrine or desamine or desfedrin or desoxo or desoxyephedrine or desoxyfed or desoxyn or destim or desyphed or detrex or dextrim or dextro desoxyephedrine or dextro methylamphetamine or dextrolevo deoxyephedrine or dextromethamphetamine or dexyfed or doe or doxephrin or doxyfed or drinalfa or effroxine or efroxinor efroxine or esophan or estimulex or eufodrin or eufodrinal or gerovit or hiropon or isophan or isophen or kemodrin or madrine or metamfetamine or metamphetamine or methamine or methampex or methamphetamin or methamphetamine or methamphetaminium or methamphin or methedrine or methoxyn or methyl amphetamine or methylamphetamine or methylbenzedrine or methylisamin or methylisomyn or methylpropamine or miller drine or methyl amphetamine or methylamphetamine or n-methylamphetamine or neopharmedrine or normadrine or norodin or norodrin or oxydess or oxydrene or oxyfed or pervitin or phenylisopropylmethylamine hydrochloride or phenylmethylaminopropane hydrochloride or philipon or philopon or premodrin or psykoton or semoxydrine or soxysympamine or syndroxor tonedron) adj2 (abus* or addict* or depend* or habituat* or misus* or mis-us* or problem* or "use disorder" or "use disorders")).tw,id. (6471)

225 (stimulant? adj2 (abus* or addict* or depend* or habituat* or misus* or mis-us* or problem* or "use disorder" or "use disorders")).tw,id. (4145)

226 ((psychostimulant? or psycho-stimulant?) adj2 (abus* or addict* or depend* or habituat* or misus* or mis-us* or problem* or "use disorder" or "use disorders")).tw,id. (2101)

227 ((stimulating or stimulative) adj (agent or agents or drug or drugs) adj2 (abus* or addict* or depend* or habituat* or misus* or mis-us* or problem* or "use disorder" or "use disorders")).tw,id. (23)

228 (cocaine adj2 (abus* or addict* or depend* or habituat* or misus* or mis-us* or problem* or "use disorder" or "use disorders")).tw,id. (25598)

229 or/224-228 (36715)

230 223 or 229 [METHAMPHETAMINE USE DISORDERS/PROBLEMATIC USE] (81568)

231 clinical trials/ (104910)

232 (randomi#ed or randomi#ation? or randomly or RCT or placebo*).tw,id. (3514398)

233 ((singl* or doubl* or trebl* or tripl*) adj (mask* or blind* or dumm*)).tw,id. (708100)

234 trial.ti. (858507)

235 or/231-234 (3862092)

236 230 and 234 [RCTs] (1562)

237 (control* adj2 trial).tw,id. (626119)

238 (nonrandom* or non-random* or quasi-random* or quasi-experiment*).tw,id. (156235)

239 (nRCT or nonRCT or non-RCT).tw,id. (895)

240 (control* adj3 ("before and after" or "before after")).tw,id. (724781)

241 time series/ (26088)

242 time series.tw,id. (73349)

243 (pretest adj3 posttest).tw,id. (22037)

244 (control* adj2 study).tw,id. (525800)

245 (pre- adj3 post-).tw,id. (284490)

246 (pretest adj3 posttest).tw,id. (22037)

247 Experiment Controls/ (915)

248 (control* adj2 group?).tw,id. (1517985)

249 or/237-248 (3105556)

250 230 and 249 [NON-RCTs] (5623)

251 cohort analysis/ (827222)

252 cohort?.tw,id. (1680684)

253 retrospective studies/ (1450969)

254 longitudinal studies/ (273650)

255 prospective studies/ (1116042)

256 (longitudinal or prospective or retrospective).tw,id. (3563731)

257 followup studies/ (708884)

258 ((followup or follow-up) adj (study or studies)).tw,id. (141027)

259 (observation$2 adj (study or studies)).tw,id. (291815)

260 ((population or population-based) adj (study or studies or analys#s)).tw,id. (45483)

261 ((comparative or comparison) adj (study or studies)).tw,id. (293087)

262 or/251-261 (6592936)

263 230 and 262 [OBSERVATIONAL STUDIES] (8009)

264 236 or 250 or 263 [ALL STUDY DESIGNS] (13334)

265 264 use medall,emczd,cctr (11029)

266 264 not 265 [PSYCINFO RECORDS] (2305)

267 77 or 165 or 199 or 266 [ALL DATABASES] (25456)

268 limit 267 to yr="2016-current" (4909)

269 remove duplicates from 268 (3339)

270 limit 267 to yr="2012-2015" (4902)

271 remove duplicates from 270 (3142)

272 limit 267 to yr="2007-2011" (5442)

273 remove duplicates from 272 (3555)

274 limit 267 to yr="2000-2006" (5728)

275 remove duplicates from 274 (3830)

276 267 not (268 or 270 or 272 or 274) (4475)

277 remove duplicates from 276 (2956)

278 269 or 271 or 273 or 275 or 277 (16822) [TOTAL UNIQUE RECORDS]

279 278 use medall [MEDLINE UNIQUE RECORDS] (7610)

280 278 use emczd [EMBASE UNIQUE RECORDS] (6992)

281 278 use cctr [CENTRAL UNIQUE RECORDS] (1596)

282 278 not (279 or 280 or 281) [PSYCINFO UNIQUE RECORDS] (624)
