# Supplementary material for: Psychosocial and pharmacologic interventions for methamphetamine addiction: protocol for a scoping review of the literature
Source: Syst Rev. 2020 Oct 24;9:245. doi: 10.1186/s13643-020-01499-z (PMC7585172; doi:10.1186/s13643-020-01499-z)
Supplement: Supplementary file 5 — Additional file 5. Draft list of data charting components. [file 13643_2020_1499_MOESM5_ESM.docx]

## Appendix 5. Draft list of data charting components

**CPGs**

- First author and year of publication
- Country of conduct
- Recommendations/key messages around treatment (with grading)
- AGREE-2 quality evaluation

**Systematic Reviews/Meta-analyses**

- First author and year of publication
- Funding source
- Date of search
- Population, Intervention, Comparison, Outcomes, Study designs, Setting (e.g., geographic or where intervention is delivered)
- Number of included studies
- Directions of estimates/message of findings around treatment, which could include summary estimates
- AMSTAR 2 quality assessment

**Primary studies**

Study characteristics, such as: First author and year of publication; Country of conduct; Study design (e.g., randomized trial, cohort study); Sample size; Inclusion and exclusion criteria; and Funding source

*Demographics of study population:*

- Sex and gender; ethnicity; employment status; income; risky behaviors (e.g., needle sharing)
- Mean/median age and age subgroups (adolescents only, adults only, mixed)
- Mental health comorbidities (e.g., psychosis; anxiety; depression; post-traumatic stress; schizophrenia; insomnia; other)
- Co-use of other substances (polypharmacy, yes/no; prescribed drugs vs alcohol/cannabis/illicit drugs; specific substances (as reported by study authors))
- History of use of methamphetamine (e.g., frequency of use in past month; use during daily life activities; administration route; measures of baseline severity of dependence and cravings)
- Subgroups (adolescents; gbMSM; sex and gender minorities; pregnant women; individuals in the corrections system; other)

*Type of intervention(s) addressed:*

- *Psychosocial:* Characteristics of interventions (e.g., frequency and duration of sessions, therapist expertise)
- *Pharmacologic*: Characteristics of interventions (e.g., drug, dose, frequency, and duration)

*Outcomes*

- **Methamphetamine use:** change in use (e.g., abstinence, reduction) including method of confirmation (self-report vs urinalysis) and mode of use.
- **Mental health effects evaluated**: changes in anxiety, depression, quality of life, suicidal behaviors, paranoia, other (as reported by study authors). Also occurrence of: psychosis, suicide, self-harm, other outcomes reported.
- **Physical effects evaluated**: sexually transmitted infections, risk behaviours (e.g., sexual risk behaviours, injection risk practices), hepatitis, endocarditis, hospitalization/emergency room visits and related traumas (e.g. concussions, motor vehicle accidents), changes in other substance use (cannabis, opioids, cocaine, hallucinogens, sedatives, hypnotics),
- **Harms**: morbidity, mortality, adverse events (AE), study withdrawal due to AEs, other outcomes reported.
- **Other effects evaluated**: Withdrawal symptoms (e.g., changes in craving, irritability/agitation, anger, sleep disturbances, appetite, headaches, joint pain, mental health effects (e.g., anxiety, depression)), self-efficacy, study retention/dropout, treatment retention/discontinuation, employment outcomes, contact with the justice system, acceptability of intervention

Definitions, timing, and format of measurement will be gathered for all outcomes.

*Conclusions*

- Summary of author interpretations/conclusions
